# *OsGRAS23*, a rice GRAS transcription factor gene, is involved in drought stress response through regulating expression of stress-responsive genes

**DOI:** 10.1186/s12870-015-0532-3

**Published:** 2015-06-13

**Authors:** Kai Xu, Shoujun Chen, Tianfei Li, Xiaosong Ma, Xiaohua Liang, Xuefeng Ding, Hongyan Liu, Lijun Luo

**Affiliations:** Shanghai Agrobiological Gene Center, Shanghai, 201106 China; Huazhong Agriculture University, Wuhan, 430070 China

**Keywords:** Drought resistance, GRAS, Rice, Transcription factor

## Abstract

**Background:**

Drought is a major abiotic stress factors that reduces agricultural productivity. GRAS transcription factors are plant-specific proteins that play diverse roles in plant development. However, the functions of a number of GRAS genes identified in rice are unknown, especially the GRAS genes related to rice drought resistance have not been characterized.

**Results:**

In this study, a novel GRAS transcription factor gene named *OsGRAS23*, which is located in a drought-resistant QTL interval on chromosome 4 of rice, was isolated. The expression of *OsGRAS23* was induced by drought, NaCl, and jasmonic acid treatments. The OsGRAS23-GFP fused protein was localized in the nucleus of tobacco epidermal cells. A trans-activation assay in yeast cells demonstrated that the OsGRAS23 protein possessed a strong transcriptional activation activity. *OsGRAS23*-overexpressing rice plants showed improved drought resistance and oxidative stress tolerance as well as less H_2_O_2_ accumulation compared with the wild-type plants. Furthermore, microarray analysis showed that several anti-oxidation related genes were up-regulated in the *OsGRAS23*-overexpressing rice plants. The yeast one hybrid test indicated that OsGRAS23 could bind to the promoters of its potential target genes.

**Conclusions:**

Our results demonstrate that *OsGRAS23* encodes a stress-responsive GRAS transcription factor and positively modulates rice drought tolerance via the induction of a number of stress-responsive genes.

**Electronic supplementary material:**

The online version of this article (doi:10.1186/s12870-015-0532-3) contains supplementary material, which is available to authorized users.

## Background

Drought is a major environmental stress factor that reduces agricultural productivity. Rice is one of the most important crops worldwide, and it consumes a large amount of fresh water resources, e.g. about 50 % in China. Developing water-saving and drought resistant rice varieties is an effective strategy to achieve food security and prevent the detrimental effects of drought and water deficit [1]. Elucidating the hereditary basis and molecular mechanism that underlies the drought resistance in rice is indispensable and vital for the development of new rice varieties with improved drought resistance [2].

Drought and water deficit can decrease photosynthetic capacity, result in oxidative damage to chloroplasts, limit metabolic reactions, and reduce dry matter accumulation and partitioning [3]. To cope with drought stress, plants have developed various strategies, which include developing larger and deeper root systems to increase water absorption from the deep soil, regulating stomata closure to reduce water loss, accumulation of compatible solutes and protective proteins, and increasing the level of antioxidants [4].

On exposure of plants to drought stresses, a series of genes are induced, the products of which would then participate in the stress responses. Transcription of these stress-response genes is largely controlled by transcription factors [5]. A number of transcription factors have been identified in the past few years that have been demonstrated to play an essential role in regulating plant responses to stresses [6]. For instance, AP2 transcription factors including DREB and CBF proteins bind to the dehydration response element and control expression of stress-responsive genes [7]. Overexpression of *DREB1B* and *DREB1A* in *Arabidopsis* enhanced freezing tolerance and dehydration/salt tolerance, respectively [8, 9]. In rice, AP2 transcription activators such as *OsDREB1A* have been isolated. *OsDREB1A* was induced by dehydration and high salinity stress [10], and overexpression of *OsDREB1A* in transgenic *Arabidopsis* improved stress tolerance. Recently, several other types of transcription factors in rice including *SNCA1* [11], *DST* [12], *MYB* [13], and *ZIP* [14, 15] have been identified to play important roles in drought resistance through regulating stomata closure, reactive oxygen species (ROS) scavenging, or other physiological processes. Although the transcription factor genes have been extensively studied, further studies are still needed to identify other novel transcription factors that are involved in stress responses.

GRAS proteins are plant specific proteins, and homologues have been found in many higher plants such as *Arabidopsis*, tomato, petunia, rice, and barley. The name is derived from the three initially identified members, *GIBBERELLIN-ACID INSENSITIVE (GAI)*, *REPRESSOR of GA1 (RGA)* and *SCARECROW (SCR)* [16]. GRAS proteins are typically composed of 400–770 amino acid residues and exhibit considerable sequence homology to each other in their respective C-terminal domains, whereas the N-terminal amino acid sequences are highly divergent [17]. GRAS family proteins are divided into several sub-families such as DELLA, SHR, SCR, PAT, LISCL, and SCL3 [18]. In the past few years, studies have demonstrated that GRAS proteins play diverse roles in gibberellin signal transduction, root development, meristem development, light signaling, biotic stress, and abiotic stress responses [19]. DELLA proteins are one of the most extensively studied GRAS subfamilies, and they function as repressors of gibberellin (GA) responsive plant growth and are key regulatory targets in the GA signaling pathway [20–22]. DELLAs have also been revealed to participate in the regulation of plant responses to jasmonic acid (JA) signaling and light signaling through interactions with the JAZ1 protein (a key repressor of JA signaling) and the light-responsive transcription factor PIFs, respectively [23–25].

In general, GRAS proteins have been hypothesized to be transcription factors; however, there are only a few reports, such as those about LISCL and NSP1/NSP2 [26–28], that show GRAS proteins acting as classic transcription factors, which have transcription activation activity and can directly bind to DNA.

There are at least 57 GRAS genes in rice, among which, several genes, such as *MOC1*, *SLR1*, *SCR*, *DLT*, and *OsGRAS19* [29–33], have been well characterized, whereas the functions of other GRAS genes in rice are unknown.

In this work, *OsGRAS23,* a GRAS gene that is localized in a rice drought resistant QTL interval, was isolated. Its expression pattern and function in rice drought resistance were investigated. It was found that *OsGRAS23* was induced by osmotic stress, and overexpression of this gene enhanced the drought resistance of transgenic rice plants. The protein possessed trans-activation activity, and it could bind to the promoter of the putative target genes. These results reveal that the OsGRAS23 protein acts as a transcription factor and is involved in the drought stress response.

## Results

### *OsGRAS23* encodes a GRAS protein that belongs to the LISCL subfamily

**Fig. 1:**
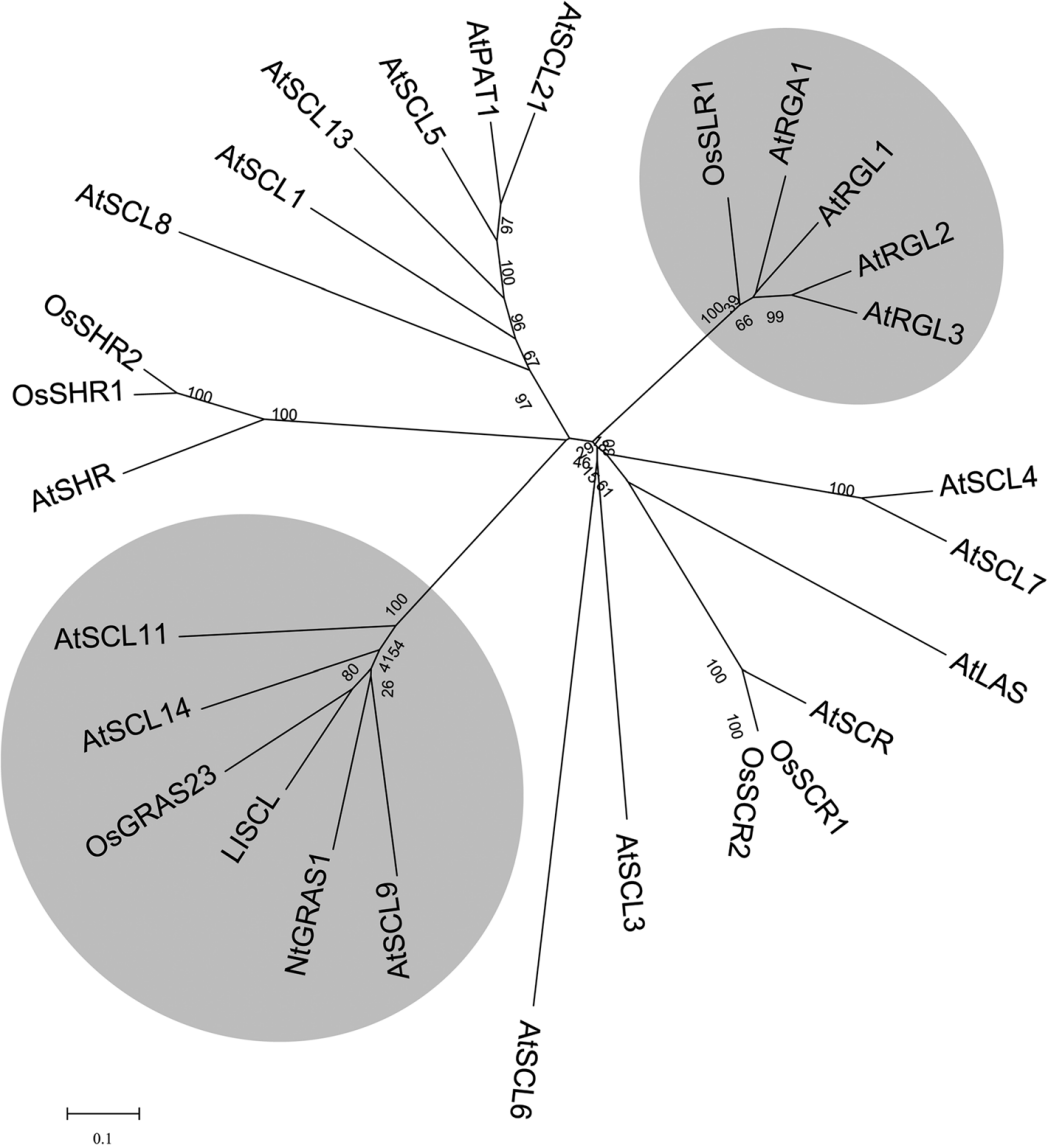
Molecular phylogenetic tree of representative members of GRAS family and OsGRAS23. Proteins are mainly from *Arabidopsis* and *Oryza sativa,* among others. The sequence alignment and phylogenetic tree construction were performed using the MEGA5 software. The DELLA and LISCL clades are indicated by gray boxes. The protein accession numbers in the Arabidopsis Information Resource (www.arabidopsis.org) and GenBank database are as follows: AtSCL1, AT1G214520; AtSCL3, AT1G50420; AtSCL5, AT1G50600; AtSCL6, AT4G00150; AtSCL7, AT3G50650; AtSCL8, AT5G52510; AtSCL9, AT2G37650; AtSCL11, AT5G59450; AtSCL13, AT4G17230; AtSCL14, AT1G07530; AtSCL21, AT2G04890; AtSHR, AT4G37450; AtSCR, AT3G54220; AtLAS, AT1G55580; AtPAT1, AT5G48150; AtRGA, AT2G01570; AtRGL1, AT1G66350; AtRGL2, AT3G03450; AtRGL3, AT5G17490; OsSHR1, A2YN56.1; OsSHR2, A2XIA8.1; OsSLR1, AC087797.5; OsSCR1, A2ZAX5.2; OsSCR2, A2ZHL0.2; OsGRAS23, NP_001053706; LISCL, BAC77269; and NtGRAS1, ABE02823.1

In our previous work, rice drought resistance QTLs were mapped using a RIL population derived from the cross between upland rice IRAT109 and paddy rice Zhenshan97B [34]. A QTL interval on chromosome 4, which contains several drought resistance QTLs, was selected for further study. Some key candidate genes in this QTL interval were chosen through bioinformatics and gene expression profile analysis [35]; among which, one candidate gene coding a GRAS transcription factor was isolated from the upland rice and identified as *OsGRAS23* (accession number: NM_001060241.1) [18]. The amino acid sequence alignment analysis showed that OsGRAS23 possesses a typical GRAS domain that included the LEUCINE HEPTAD, VHIID, PFYRE, and SAW motifs in its C-terminus (Additional file 1: Figure S1). Phylogenetic analysis indicated that OsGRAS23 groups with the LISCL branch contained *Arabidopsis* homologs AtSCL9 and AtSCL14 (Fig. 1).

### Expression pattern of *OsGRAS23*

**Fig. 2:**
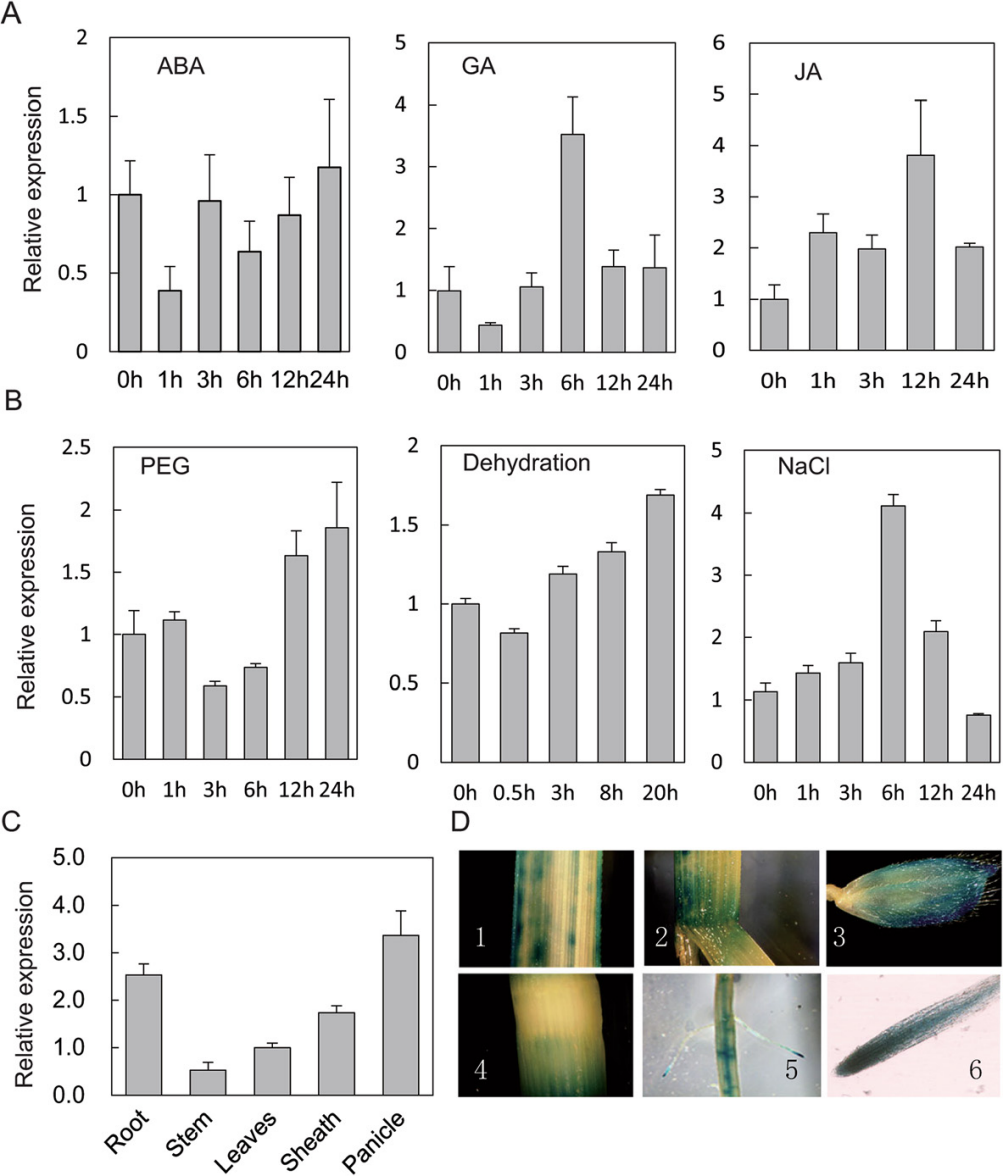
Expression patterns of *OsGRAS23*. **a** Relative expression level of *OsGRAS23* under hormone treatment including ABA (100 μM), GA (100 μM), and JA (100 μM). **b** Relative expression level of *OsGRAS23* under stresses include PEG6000 (20 %), dehydration, and NaCl(100 mM). **c** Relative expression of *OsGRAS23* in different tissues (root, stem, leaves, sheath and panicles) of rice plants under normal condition. All the expression levels of *OsGARS-23* were examined by quantitative real-time PCR. The data represent the mean ± SE (n = 3). **d** OsGRAS23 promoter: *GUS* expression pattern in transgenic rice plants. GUS staining in the leaves, sheath (2), spike (3), stem (4), root (5), and root tip (6)

To investigate and predict the function of *OsGRAS23*, its expression patterns under various stresses and phytohormone treatments were investigated by real-time quantitative RT-PCR. As shown in Fig. 2, the expression of *OsGRAS23* was significantly induced by polyethylene glycol (PEG), dehydration, salt, GA, and JA treatment in the rice leaves.

The tissue expression pattern of *OsGRAS23* was further investigated by transforming rice with a β-glucuronidase (GUS) construct driven by the *OsGRAS23* promoter. After staining the transgenic rice plants, strong GUS signals were detected in the root tips and spikes. There were also GUS signals in the stem and leaves, but the signals were relatively weak (Fig. 2D). Real-time PCR further confirmed that *OsGRAS23* expression was higher in rice panicles and roots than in the stems and leaves (Fig. 2C).

### Molecular characterization of *OsGRAS23-*overexpressing transgenic rice plants

**Fig. 3:**
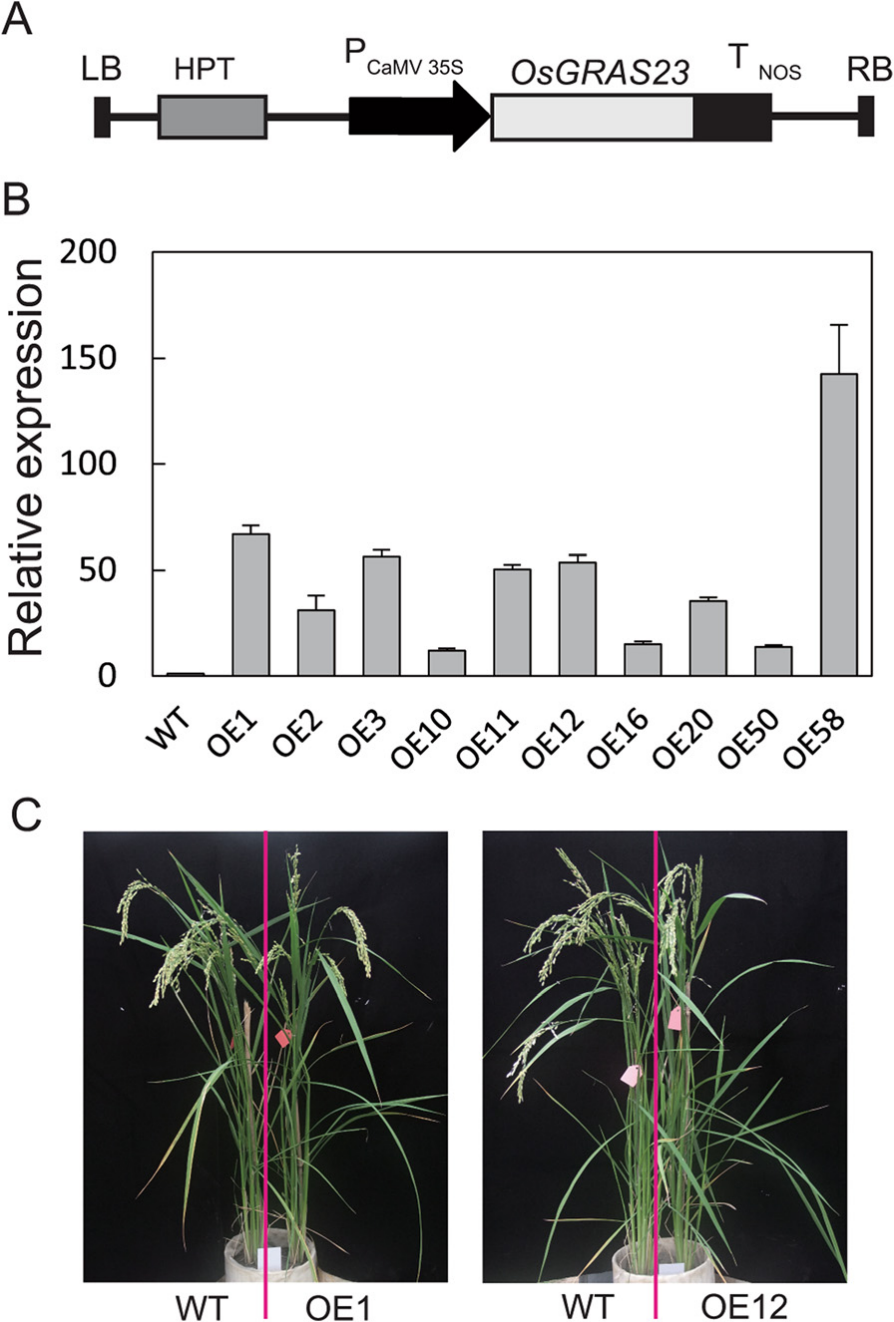
Molecular identification of *OsGRAS23*-overexpressing transgenic lines. **A** Schematic diagram of overexpression construct. LB, left border; HPT, hygromycin phosphotransferase; P_CaMV35S_, cauliflower mosaic virus 35S promoter; T_NOS_, nopaline synthase gene terminator; and RB, right border. **B** Relative expression levels of *OsGRAS23* in transgenic rice lines. WT: wild type, OE1-OE58: transgenic lines. The gene expression level was analyzed by quantitative real-time PCR. The data represent the mean ± SE (n = 3). **C** Phenotypes of transgenic rice plants and WT plants grown in PVC pipes

To investigate the biological function of *OsGRAS23*, transgenic rice plants over-expressing *OsGRAS23* under the control of the constitutive CaMV35S promoter were produced (Fig. 3A). Real-time PCR analysis showed that the expression levels of *OsGRAS23* in these transgenic rice plants were significantly higher than in the wild type (WT) Zhonghua11(ZH11) plants (Fig. 3B). No significant differences in morphological (Fig. 3C) and agronomic traits (data not shown) between the WT and transgenic plants in the adult plant stage were observed.

### Overexpression of *OsGRAS23* improved tolerance of transgenic rice plants to drought and oxidative stresses

To further determine the function of OsGRAS23 on the plant stress responses, various stress treatments on the transgenic rice and WT plants were performed.

**Fig. 4:**
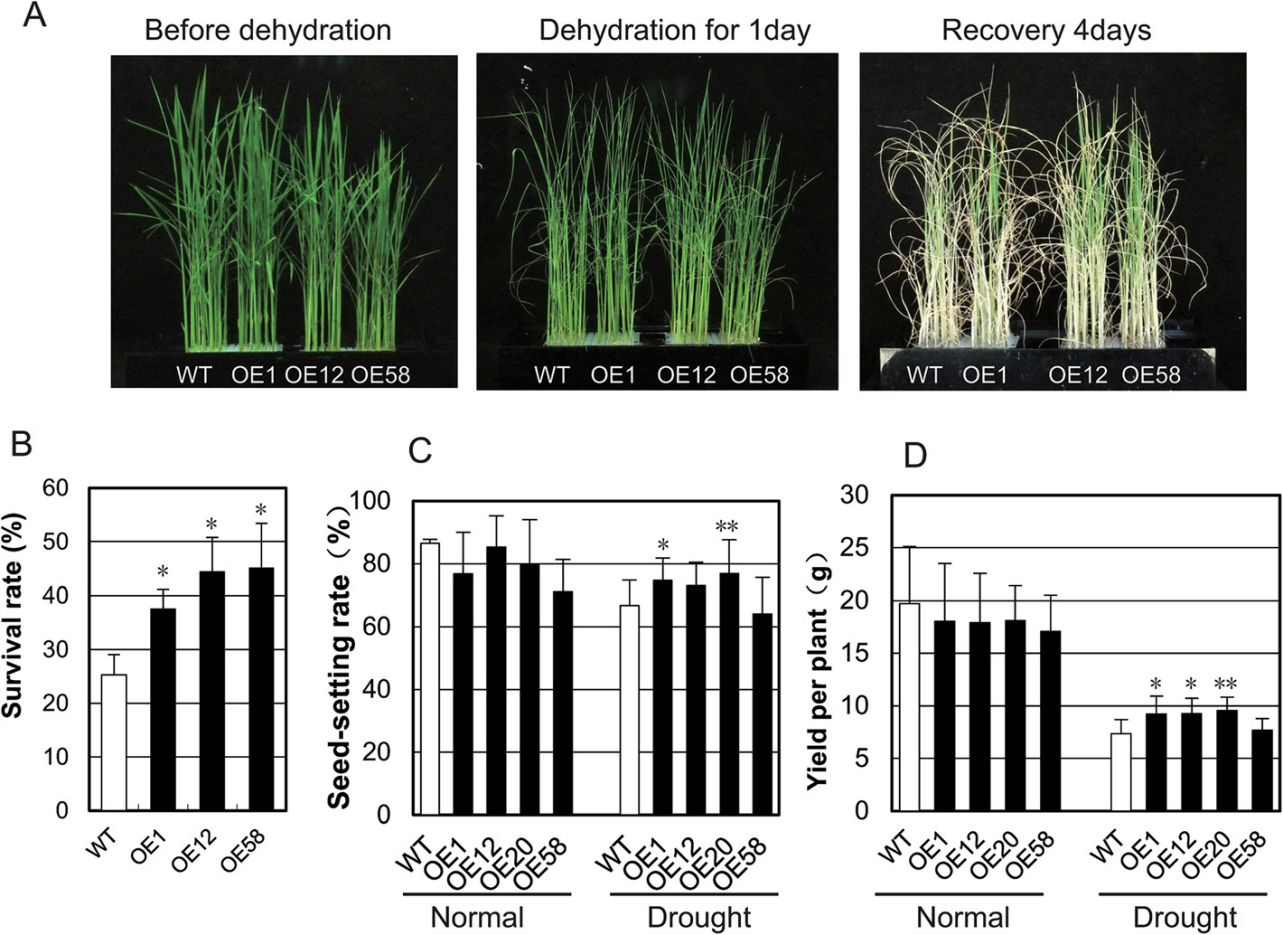
Drought resistance assay of *OsGRAS23*-overexpressing transgenic rice. **a** Three-week-old transgenic rice plants and WT plants grown in the 96-well plates and cultivated with culture solution, and exposed to dehydration stress for one day before being transferred to the culture solution. **b** Survival rate of WT and transgenic lines after dehydration stress. The data represent the mean ± SD (n = 3),*P ≤ 0.05. **c**, **d** Seed setting rate and yield of transgenic rice plants under drought stress at the reproductive stage. The data represent the mean ± SD (n = 8),*P ≤ 0.05, ** P ≤ 0.01, *t*-test

Under the dehydration stress condition, *OsGRAS23*-overexpressing rice plants showed a lower degree of wilting and better recovery compared with WT plants (Fig. 4A), and 37-45 % of *OsGRAS23*-overexpressing plants recovered, which was significantly more than that of the WT plants (Fig. 4B). When grown in a paddy field under drought stress during the later tillering stage, the seed setting rate and yield of *OsGRAS23*-overexpressing plants (OE1 and OE20 lines) were significantly higher than those of the WT plants (Fig. 4C and D). These results indicated that overexpression of *OsGRAS23* could improve the drought resistance of the transgenic rice plants.

**Fig. 5:**
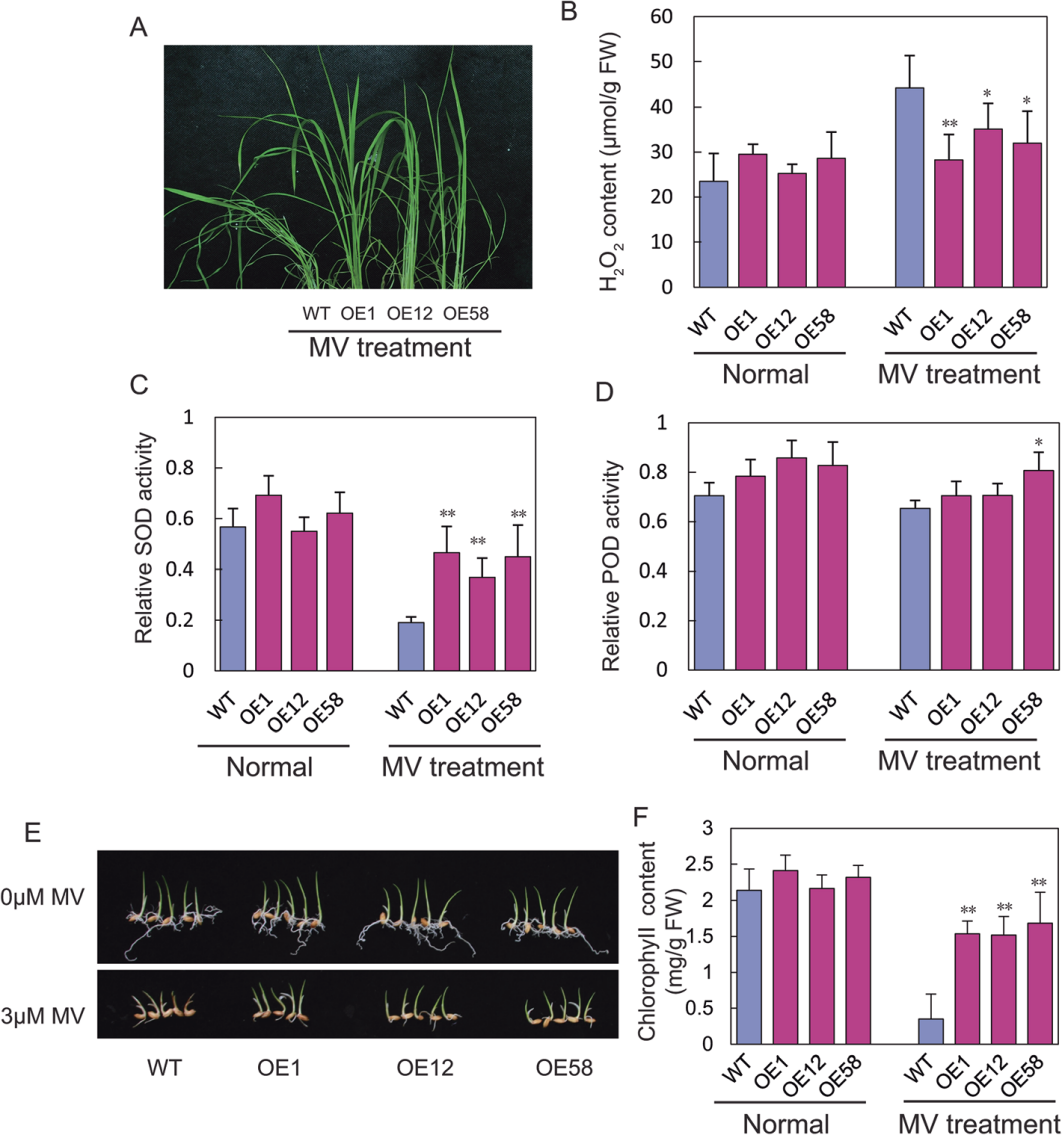
Overexpression of *OsGRAS23* improved tolerance to oxidative stress. **a** Overexpression and WT rice plants were grown with MV. Total H_2_O_2_ content (**b**), relative SOD activity (**c**), and relative POD activity (**d**) in the leaves of the transgenic and WT rice plants were measured before MV treatment and after the plants were treated with 30 μM MV for 24 h. Overexpression and WT rice seeds were grown with 3 μM MV (**e**) and the total chlorophyll contents were measured in the leaves (**f**). The data represent the mean ± SD (n = 4 or 5), *P ≤ 0.05, ** P ≤ 0.01, *t*-test, FW: fresh weight

The seedlings of the WT and *OsGRAS23*-overexpressing plants were also treated with 30 μM methyl viologen (MV), which is an oxidative stress inducer, to determine the tolerance to oxidative stress. After treatment for 24 h, the WT rice plants had wilting that was more severe than the *OsGRAS23*-overexpressing plants (Fig. 5A). Physiological analysis showed that under the MV treatment, the transgenic rice plants accumulated less H_2_O_2_ and possessed higher superoxide dismutase (SOD) and peroxidase (POD) activities compared with the WT plants (Fig. 5C and D). Similarly, after 3 μM MV treatment for four days, the WT plants showed a much more severe etiolating phenotype than the OsGRAS23-overexpressing plants (Fig. 5E), and the chlorophyll content in the leaves of the WT plants was significantly lower than that of the transgenic plants (Fig. 5F). These results demonstrated that the overexpression of *OsGRAS23* enhanced ROS scavenging ability and tolerance to oxidative stress of the transgenic rice plants.

### OsGRAS23, which had transcription activation activity in yeast cells, localized in cell nucleus of tobacco epidermal cells

**Fig. 6:**
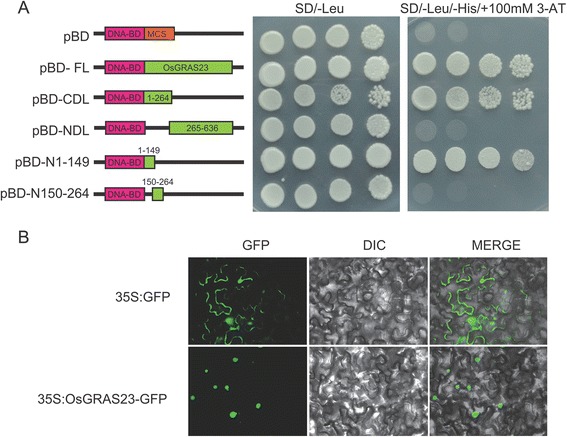
Trans-activation assay and sub-cellular localization of OsGRAS23. **a** Trans-activation activities of different portions of OsGRAS23 were checked in yeast MaV203. BD: GAL4 DNA binding domain; FL: full length; CDL: C-terminus deleted domain; and NDL: N-terminus deleted domain. **b** Sub-cellular localization of OsGRAS23. GFP and OsGRAS23-GFP fusion gene under the control of the CaMV 35S promoter separately expressed transiently in the tobacco epidermal cells

LISCL was reported to be localized in the cell nucleus and had transcription activation activity in yeast and plant cells [26]. To determine the sub-cellular localization of the OsGRAS23 protein, a chimeric gene comprised of OsGRAS23 coding region and green fluorescent protein (GFP) under the control of the CAMV35S promoter was constructed. *Agrobacterium tumefaciens* carrying the OsGRAS23-GFP or GFP constructs were infiltrated into tobacco leaves, and the GFP was observed at two days after agroinfiltration. The tobacco epidermal cells transformed with an empty GFP vector alone displayed fluorescence throughout the cell. However, in cells expressing the OsGRAS23–GFP construct, fluorescence was found to be localized in the nucleus (Fig. 6B). The result indicated that the OsGRAS23 protein is a nuclear-localized protein.

To assess the function of the OsGRAS23 protein as a transcription factor, the full length of OsGRAS23 was fused to the DNA binding domain of GAL4 and introduced into yeast MaV203 cells. As Fig. 6A shows, the full length of OsGRAS23 could strongly induce the expression of the reporter genes, which indicated that the OsGRAS23 protein had transcription activation activity. Motifs of OsGRAS23, which are responsible for the transcription activation, were further characterized through checking the transcription activation activities of different partial fragments of OsGRAS23 in yeast. Consistent with the previous proposition [17], the relative conserved motif (NI) in the N terminal of OsGRAS23 had strong trans-activation activity; whereas, the C-terminal GRAS domain showed no obvious trans-activation activity (Fig. 6A). The results revealed that the OsGRAS23 protein is a putative transcription factor and that the N-terminal region of OsGRAS23 is required for transcription activation.

### OsGRAS23 regulated stress-response genes and binding to promoters of several putative target genes

**Fig. 7:**
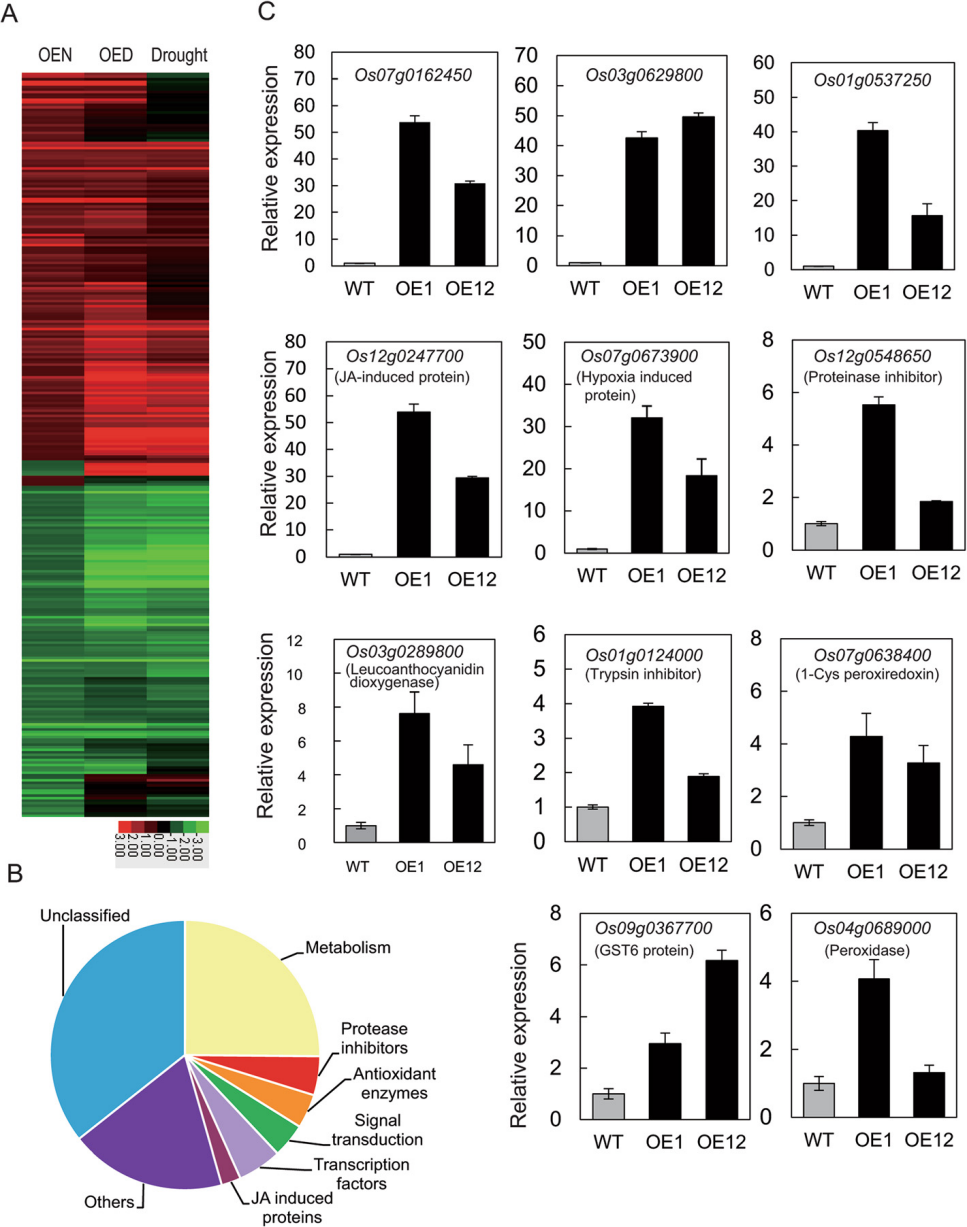
Gene expression profile analysis of *OsGRAS23*-overexpressing transgenic rice plants. **a** Drought responsive expression pattern of all differently expressed genes in transgenic plants. OEN: genes differently expressed between *OsGRAS23*-overexpressing transgenic rice plants and WT plants under normal condition. OED: genes differently expressed between OsGRAS23-overexpressing transgenic rice plants and WT plants under dehydration treatment. Drought: genes differently expressed in the WT plants between dehydration treatment and normal condition. **b** Classification of up-regulated genes in the transgenic plants compared with the WT plants. **c** Relative expression levels of some up-regulated genes in transgenic rice plants. qRT-PCR was used to analyze the expression levels. The data represent the mean ± SE (n = 3)

To search the downstream genes of OsGRAS23, the gene expression profiles of *OsGRAS23*-overexpressing plants and WT plants were analyzed using the Affymetrix Rice Genome Genechip. It was found that 175 genes were up-regulated (fold > 2) and 160 genes were down-regulated (fold < 0.5) in the *OsGRAS23*-overexpressing plants compared with the WT plants under normal condition (Additional file 2: Table S1). Among the up-regulated genes, 76 genes were also induced by drought stress in the WT rice plants, which suggests that these up-regulated genes by OsGRAS23 may participate in the drought response (Fig. 7A). These up-regulated genes encode proteins with diverse functions such as transcription factors, JA induced proteins, protease inhibitors and antioxidant enzymes (Fig. 7B).

To further confirm the microarray results, the transcription levels of several up-regulated genes were analyzed using qRT-PCR. The results were consistent with the microarray results. The expression of the obviously up-regulated genes (i.e. *Os07g0162450, Os03g062980*, and *Os01g0537250*) was highly induced in *OsGRAS23*-overexpressing lines. The expression of the other genes related to anti-oxidation and defense responses (e.g. *Os04g068900* encoding peroxidase; *Os07g0638400* encoding peroxiredoxin; *Os09g036770* encoding glutathione-s-transferase (GST); *Os03g0289800* encoding leucoanthocyanidin dioxygenase; and *Os12g0548650* and *Os01g0124000* encoding proteinase inhibitors) in *OsGRAS23*-overexpressing lines was also significantly higher than that of the WT plants (Fig. 7C). This suggested that the transcription of these genes may be positively regulated by OsGRAS23.

**Fig. 8:**
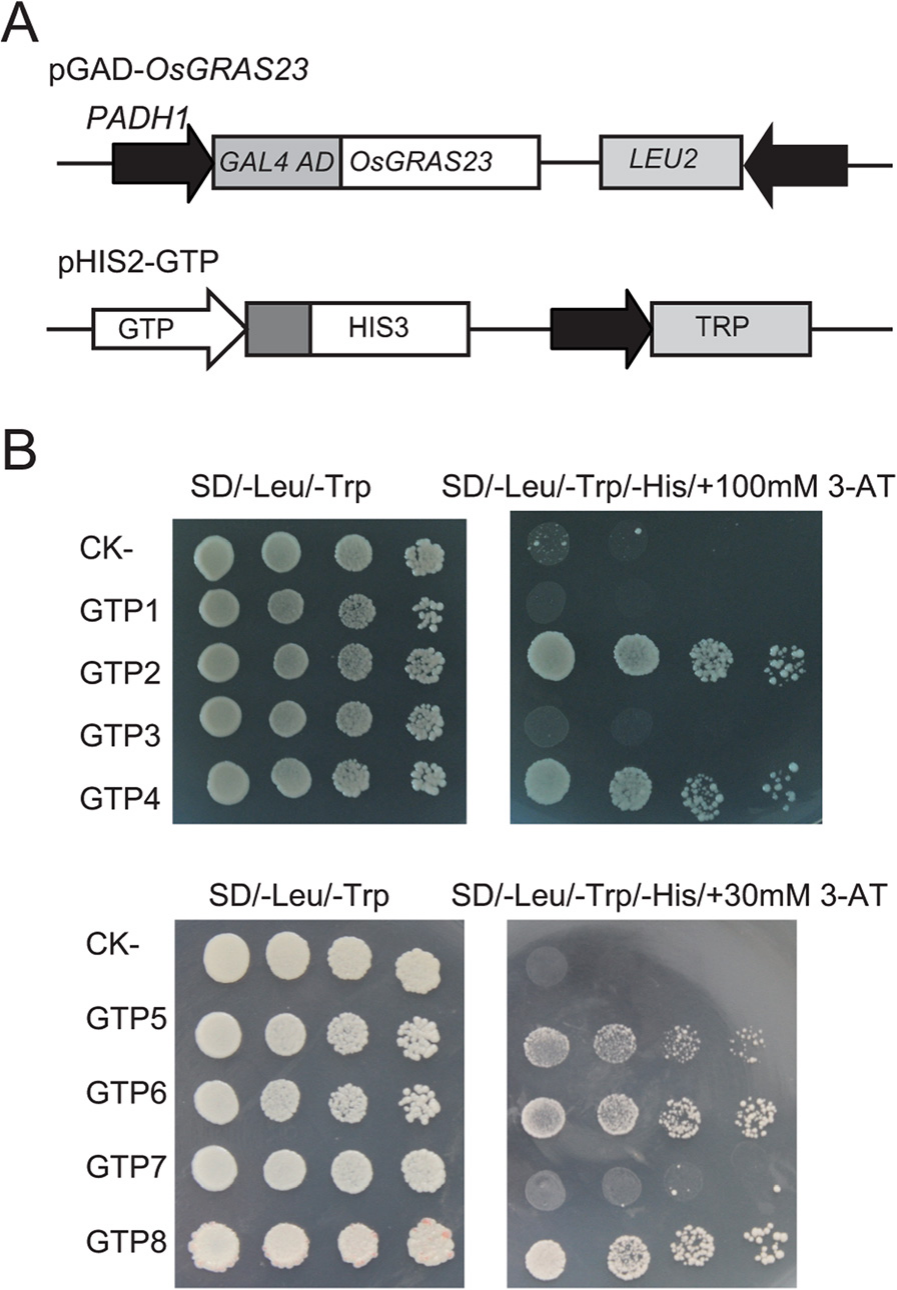
Identification of putative target genes regulated by OsGRAS23 with yeast one hybrid assay. **a** Schematic structure of yeast expression construct pGAD-OsGRAS23 and reporter construct pHIS2.1-GTP (OsGRAS23 putative target gene promoter). **b** Growth performance of transformants on SD/-Leu-/Trp/-His medium containing 100 mM or 30 mM 3-AT. GTP1-GTP8 indicates the pGAD-OsGRAS23 plus pHIS2.1-cis (promoters of *Os07g0162450*, *Os01g0537250*, *Os07g0638400*, *Os03g0629800, Os04g0173800, Os03g0289800, Os06g0513781*, *and Os07g0673900* in pHIS2.1, respectively). ck-: negative control (pGADT7-rec2-OsGRAS23 plus p53HIS2.1)

Based on the microarray analysis, eight up-regulated genes were selected to confirm whether the OsGRAS23 protein could bind to the promoter region of these genes via the yeast one hybrid assay. The pGAD-OsGRAS23 plasmid (containing the putative DNA-binding domain of OsGRAS23 fused to the GAL4 activation domain) and the reporter construct pHIS-cis (1.2 kb promoters of the eight putative target genes) were co-transformed into yeast strain Y187(Fig. 8A). As indicated by the activation of the reporter genes, OsGRAS23 can bind to the promoters of several genes (*Os03g062980*, *Os01g0537250*, and *Os07g0673900* encoding hypoxia induced protein; *Os04g0173800* encoding lectin precursor; and *Os03g0289800*encoding leucoanthocyanidin dioxygenase) (Fig. 8B). These results implied that OsGRAS23 has DNA binding activity and may directly regulate the expressions of these target genes.

## Discussion

### OsGRAS23 encodes drought-responsive GRAS protein

The transcriptional regulation of stress related genes is one of the crucial steps during plant stress responses, and transcription factors play important roles in these processes [36]. For example, osmotic stress activates several transcription factors, including NAC proteins, which activate an early response to dehydration1 (ERD1) [37]. Several bZIP proteins have been characterized to be ABA -responsive transcription factors (AREB/ABF) that bind to the ABREs and have a pivotal role in ABA-dependent gene activation [38–40]. There remain a number of transcription factors, especially novel type transcription factors, that have not been studied in detail. In this study, the OsGRAS23 protein was characterized to be a novel GRAS transcription factor that is involved in rice abiotic stress responses.

GRAS proteins are plant specific proteins, for which a large number of reports have shown that they play important roles in plant growth, development and phytohormone signal transduction. However, reports referring to GRAS proteins being involved in abiotic stress were relatively few. In this study, *OsGRAS23* was isolated from rice and shown to belong to the LISCL subfamily, and it is an ortholog to NtGRAS1, SCL9, and SCL14 (Fig. 1). Previous research showed that the expression of these genes was induced by abiotic stresses [41, 42]. SCL14 has been found to play an important role in plant stress response [43]. The close genetic relationship between OsGRAS23 and these proteins suggests that OsGRAS23 might also play a role in plant abiotic stress responses.

The expression of *OsGRAS23* was induced by drought stress (Fig. 2). The hormones JA and GA but not ABA, can also induce the expression of *OsGRAS23*. Furthermore, the promoter of *OsGRAS23* contained the heat stress responsive element, cis-acting regulatory element involved in MeJA-responsiveness, GA-responsive element, and other cis-elements involved in stress and phytohormone responsiveness, which were predicted using PlantCARE (data not shown). These results implied that OsGRAS23 is a stress-responsive GRAS protein that may be involved in plant responses to abiotic stresses (e.g. drought) and phytohormone signaling (e.g. JA).

### OsGRAS23 protein functioned as a GRAS transcription factor

Some of the previously characterized GRAS proteins functioned through interactions with other proteins in the signal transduction pathway, and they were found to participate in transcription regulation. The regulations could be divided into two types: transcription co-regulator or transcription factor [17]. When functioning as transcription factors, several GRAS proteins, such as LISCL and NSP, have both trans-activation activities and the ability to directly binding to DNA which was indicated by assays in yeast or plant cells [26, 27, 44]. In the current work, the genetic relationship of the OsGRAS23 protein and LISCL protein was close (Fig. 1), which suggests that the OsGRAS23 protein may also act as a transcription factor. Further characterization indicated that the OsGRAS23 protein was mainly localized in the cell nucleus, and that it showed obvious trans-activation activity and DNA binding ability in yeast cells (Fig. 6 and Fig. 8). Moreover, several genes were up-regulated in *OsGRAS23*-overexpressing rice plants, which supports that OsGRAS23 could positively modulate the expressions of down-stream genes (Fig. 7), these results confirmed the hypothesis that OsGRAS23 functions as a transcription factor.

The expression of several genes in transgenic rice plants was obviously higher than that of the WT rice (Fig. 7), and this strongly suggested these genes may be directly regulated by OsGRAS23. The yeast one hybrid assay further confirmed the OsGRAS23 protein could directly bind to the promoters of several up-regulated genes, such as *Os03g0289800* which encodes leucoanthocyanidin dioxygenase (Fig. 8B). However, it was found that OsGRAS23 could not bind to the promoters of the genes encoding ROS scavenging enzymes (e.g. *Os07g0638400*), which implies that these genes related to anti-oxidation activity were indirectly regulated by OsGRAS23.

The GRAS protein generally contains the conserved GRAS domain in the C-terminus, whereas the N-terminus was relatively disordered. Previous bioinformatics research proposed that the motif richness in acidic residues flanking the repeated hydrophobic/aromatic residues in the N-terminus might be associated with transcription activation [17]. While alternative previous reports referred to the N terminus of NSP1/NSP2 and LISCL as being the main trans-activation domain [26, 27]. In this study, the N-terminus domain of OsGRAS23 was also responsible for the trans-activation activity. Further analysis found that the main trans-activation motif was the first conserved motif (NI) but not the second motif (NII) (Fig. 6A), which is similar to the LISCL protein.

### OsGRAS23 positively regulated rice drought tolerance through upregulating genes related to stress responses

A few studies have found that the GRAS proteins are involved in abiotic stress responses. *PeSCL7* was recently isolated from *Populus euphratica Oliv*, and its overexpression in *Arabidopsis* showed improved drought and salt tolerance [45]. *NtGRAS1* was cloned from tobacco and shown to belong to the LISCL subfamily, and its expression was induced by drought, salt, and H_2_O_2_ treatments [41]. DELLA proteins are also involved in the ROS reaction [46] and development coordination during abiotic stress [47]. Here, overexpression of *OsGRAS23* conferred enhanced resistance to drought stress and oxidative stress on transgenic rice (Fig. 4 and Fig. 5), which supplies novel evidence for GRAS proteins functioning in rice abiotic stress responses.

As a putative transcription factor, OsGRAS23 may participate in plant responses to stress through regulating the transcription of downstream genes. Microarray analysis showed that a number of drought-induced genes were indeed up-regulated in the OsGRAS23-overexpressing rice plants (Fig. 7A and Additional file 2: Table S1). The up-regulated genes encode both regulatory and functional proteins, such as transcription factors, protein kinases, anti-oxidants, proteinase inhibitors, and enzymes related to metabolism (Fig. 7B and Additional file 2: TableS 1). The homologues of these genes were reported to play roles in plant stress tolerance. For instance, ROS scavenging enzymes, including peroxiredoxin, peroxidase, and glutathione-S-transferase, have been verified to be responsible for alleviating oxidative damage and enhancing plant stress tolerance [48–51]. The activities of ROS scavenging enzymes (SOD and POD) were increased and accumulated H_2_O_2_ was reduced in the OsGRAS23-overexpressing plants under oxidative stress (Fig. 5), which further suggests that the enhanced ROS scavenging ability in the transgenic plants might partly contribute to the enhanced drought tolerance of the transgenic plants. Proteinase inhibitors, such as Bowman Birk trypsin inhibitors, were also revealed to confer plant stress tolerance probably through inhibiting the degradation of the stress-mitigating protein [52, 53]. Leucoanthocyanidin dioxygenase is involved in the biosynthesis of anthocyanin which is one class of flavonoids [54]; previous studies have shown that flavonoids are associated with plant stress adaptation [55]. It was also noticed that some genes that were up-regulated in the transgenic plants encoded lectin precursors, protease inhibitors, and JA induced proteins, which suggests that OsGRAS23 might also be involved in the defense responses mediated by JA. Taken together, OsGRAS23 increases transcription of genes related to the stress responses (especially antioxidant and protein protection) and positively regulates rice drought tolerance.

## Conclusions

We isolated a rice GRAS gene, *OsGRAS23,* from a rice drought resistance QTL interval and characterized its function. Drought, NaCl, JA, and GA treatments induced the expression of *OsGRAS23*. The OsGRAS23 protein was localized in the nucleus and possessed a strong transcriptional activation activity. Furthermore, the OsGRAS23 protein could bind to the promoters of several target genes and modulated the expressions of a series of stress-related genes. Overexpression of *OsGRAS23* conferred transgenic rice plants with improved drought resistance. We can therefore conclude that *OsGRAS23* encodes a novel stress-responsive GRAS transcription factor and positively regulates the rice drought stress response.

## Methods

### Plant material, stress treatment, and gene expression pattern analysis

To analyze the expression pattern of *OsGRAS23*, seedlings of the upland rice cultivar IRAT109 (*Oryza sativa L. ssp japonica*) at the four leaf stage were treated with 20 % (m/v) PEG6000, dehydration, and 100 mM NaCl, and then sampled at the designated times. For the phytohormone treatment, 0.1 mM ABA, JA and GA were separately sprayed on to the seedlings while the roots were also submerged into the solution.

Total RNA was extracted using the TRNzol reagent (TIANGEN), and cDNA was synthesized by PrimerScript reverse transcriptase (TaKaRa). Real time quantitative PCR were performed in 96-well plate with a Bio-Rad CFX96 Real-Time PCR Detection System (Bio-Rad) using the SYBR premix Ex Taq (TaKaRa). The reaction procedure was as follows: 95 °C for 60s, followed by 40 cycles at 94 °C for 15 s and 62°Cfor 60s. The rice actin gene was used as the reference gene to normalize the target gene expression, which was calculated using the relative quantization method (2^-ΔΔCT^).

### Vector construction and rice transformation

The full-length cDNA of *OsGRAS23* was amplified from the cDNA of upland rice IRAT109, and then it was cloned into the pMD-18 T vector for sequencing. The primers used in this study are listed in Additional file 3: Table S2. The GRAS protein sequence alignment was performed using Clutal W, and a phylogenetic tree was constructed using the neighbor joining method of MEGA5.1. The full-length cDNA of *OsGRAS23* was digested with *Xba*I and *BstE*II, and then ligated into the plant expression vector pCAMBIA1323, which was digested with the same enzymes. Thus, *OsGRAS23* was driven by the CaMV35S promoter.

The 1.3 kb promoter sequence upstream of *OsGRAS23* predicted ATG codon was isolated from the genome DNA of IRAT109. For tissue expression pattern analysis, the promoter was ligated upstream of the GUS reporter gene in pBI121 after digestion with *Bam*HI and *Kpn*I.

Both of the constructs were introduced into the *Japonica* rice Zhonghua11 (ZH11) via the *A. tumefaciens*-mediated transformation method. The transgenic rice plants were selected on Murashige and Skoog (MS) medium containing hygromycin. The transgenic rice plants were primarily characterized through PCR to confirm whether *OsGRAS23* had been successfully integrated into the rice genome.

To investigate the *OsGRAS23* expression pattern in tissues, the positive Pro_OsGRAS23_:GUS transgenic rice plants were sampled and stained using a histochemical staining method described previously [56].

To test whether *OsGRAS23* was highly expressed in the *OsGRAS23*-overexpressing rice plants, real-time quantitative RT-PCR was performed, and the expression levels of *OsGRAS23* in the transgenic rice were calculated as described above.

### Sub-cellular localization

To investigate the sub-cellular localization of the OsGRAS23 protein, the full-length of *OsGRAS23* was cloned into the plant expression vector pCAMBIA1300EGFP after being digested by *Xba*I and *Bam*HI, which enabled OsGRAS23 to be fused with GFP. The GFP fusion vector was transformed into *A. tumefaciens* strain EHA105 and the transformed *Agrobacterium* were infiltrated into leaves of *Nicotiana benthamiana* plants as described previously [57]. These agroinfiltrated plants were allowed to grow for 48 h and the GFP fluorescence was examined under a Leica fluorescence microscope.

### Trans-activation assay in yeast cells

The trans-activation activity of OsGRAS23 was investigated in yeast cells. The full-length, N-terminus truncated fragment and C-terminus truncated fragment of *OsGRAS23* were separately fused into the frame with the yeast GAL4 DNA-binding domain in the vector pDEST32 by the recombination reaction (Invitrogen). The different constructs were individually introduced into the yeast strain MaV203 (MATα; leu2–3,112; trp1–901; his3D200; ade2–101; gal4D; gal80D; SPAL10::URA3; GAL1::lacZ; HIS3UASGAL1::HIS3@LYS2; can1R; cyh2R; Invitrogen) using the lithium acetate method and the transformants were selected on SD/-Leu media. The positive transformants were assessed through a seral dilution on the SD/-Leu/-His medium supplemented with different concentrations of 3-amino-1,2,4-triazole(3-AT).

### Stress treatments of plant material and physiological measurement

For all the stress treatments, the seeds of the T3 overexpression lines were germinated on the MS medium supplement with 50 mg/L hygromycin and WT seeds were grown in the MS medium. For the dehydration treatment, the most uniformly germinating seeds were sown in a 96-well plate from which the bottom had been removed. The seedlings were grown in liquid culture solution in a growth chamber with a 16 h light (28 °C)/8 h dark(24 °C) photoperiod/temperature rotation. Then, 21 day-old seedlings were transferred onto filter paper to induce dehydration stress. After the WT plants wilted, all the plants were transferred into the normal culture solution.

Drought stress testing at the panicle development stage (~two weeks before flowering) was performed in a paddy field in a greenhouse. Drought stress was initiated and developed by stopping the supply of water until all of the leaves became rolled (wilted), and the rice plants were recovered with re-irrigation.

### Oxidant stress treatment and ROS related biochemical assay

The T3 transgenic lines were sown in 96-well plates and grown in culture solution as described above. After this, 21 day-old seedlings were transferred into the culture solution supplemented with 30 μM methyl viologen (MV) and grown for 24 h. The leaves were harvested for ROS related biochemical analysis.

H_2_O_2_ was extracted from leaves according to a method described previously [58], and quantitative measurement of H_2_O_2_ production was performed using the Amplex Red hydrogen peroxide/peroxidase assay kit (Molecular Probes) following the manufacturer’s instructions. To measure the activity of ROS-scavenging enzymes, soluble proteins were extracted using physiological salt buffer, and the activities of SOD, POD, and catalase (CAT), were measured using a kit from Nanjing Jiancheng Bioengineering Institute (Jiangsu, China).

For another oxidative stress test, the germinated seeds of WT and transgenic lines were transferred into the culture solution containing 3 μM MV and grown for four days. Chlorophyll content was then measured as describe previously [59].

### Micro-array analysis, qRT-PCR, and yeast one hybrid

The 21-day-old seedlings of WT and OsGRAS23-overexpressing rice plants were harvested for micro-array analysis. Half of the plants were sampled as untreated controls, and the other half of the plants were transferred onto filter paper to induce drought stress, and when the leaves of the WT plants began to roll, they were collected for RNA isolation. Two biological replicates (20 seedlings per replicate) were performed. The total RNA was extracted using the TRizol reagent. For micro-array analysis, the experimental procedure followed the standard protocol of the Affymetrix GenChip service (Gene biotech). The data were analyzed with the Robust Multichip Analysis (RMA) algorithm using the default analysis settings and global scaling as the normalization method by Parterk Genomics Suite 6.5. The differentially expressed genes between the transgenic and WT rice plants that had fold changes higher than two (up-regulated) or less than 0.5 (down-regulated) were selected. The expression of some obviously up-regulated genes was confirmed through real-time quantitative RT-PCR.

Among the up-regulated genes, eight genes (i.e.*Os07g0162450*, *Os01g0537250*, *Os03g0629800*, *Os07g0638400*, *Os06g0513781*, *Os07g0673900*, *Os04g0173800*, and *Os03g0289800*) were chosen for further analysis. The upstream 1.2 kb promoters of these possible target genes were cloned from the genomic DNA of IRAT109. For the yeast one hybrid, the promoter sequences were cloned into the yeast expression vector pHIS2.1 between the *Sma*I and *EcoR*I sites. The constructs were co-transformed into the yeast Y187 (MATa; ura3–52; his3–200; ade2–101; trp1–901; leu2–3, 112; gal4D; gal80D; met–; URA3::GAL1 UAS-GAL1 TATA-LacZ; MEL1; Clontech) with the pGADT7-Rec2-*OsGRAS23* vector which produced a fusion protein of OsGRAS23 and GAL4 trans-activation domain. The DNA-protein interactions were determined by the growth of the transformants on SD/-Leu/-Trp/-His plates supplied with different concentrations of 3-AT.

### Availability of supporting data

The microarray data supporting the results of this article are available in NCBI Gene Expression Omnibus repository (http://www.ncbi.nlm.nih.gov/geo/) under accession number GSE64576.

Data for the phylogenetic analysis can be found in TreeBASE (http://purl.org/phylo/treebase/phylows/study/TB2:S17663)

## Additional files

Additional file 1: Figure S1. Alignment of deduced amino acids of OsGRAS23 with the well-characterized GRAS family proteins. The lines above the alignment indicate the locations of proposed trans-activation motifs (NI and NII) and the conserved regions in the GRAS proteins were defined previously. The protein accession numbers are as follows: AtSCL9, AT2G37650; AtSCL14, AT1G07530; AtSHR, AT4G37450; AtLAS, AT1G55580; AtPAT1, AT5G48150; AtRGA1, AT2G01570; and OsSLR1, AC087797.5. Additional file 2: Table S1. Micro-array analysis of *OsGRAS23*-overexpressing rice plants. Genes that were up-regulated greater than 2-fold or down-regulated lower than 0.5 in overexpression lines compared with WT are listed. Additional file 3: Table S2. Primers used in this research.

## Abbreviations

GA: Gibberellin
JA: Jasmonic acid
ROS: Reactive oxygen species
SOD: Superoxide dismutase
POD: Peroxidase
GST: Glutathione-s-transferase
MV: Methyl viologen
GUS: β-glucuronidase
WT: Wild type
OE: Overexpression
