# Supplementary material for: OsGRAS23, a rice GRAS transcription factor gene, is involved in drought stress response through regulating expression of stress-responsive genes
Source: BMC Plant Biol. 2015 Jun 13;15:141. doi: 10.1186/s12870-015-0532-3 (PMC4465154; doi:10.1186/s12870-015-0532-3)
Supplement: Additional file 1: Figure S1. — Alignment of deduced amino acids of OsGRAS23 with the well-characterized GRAS family proteins. The lines above the alignment indicate the locations of proposed trans-activation motifs (NI and NII) and the conserved regions in the GRAS proteins were defined previously. The protein accession numbers are as follows: AtSCL9, AT2G37650; AtSCL14, AT1G07530; AtSHR, AT4G37450; AtLAS, AT1G55580; AtPAT1, AT5G48150; AtRGA1, AT2G01570; and OsSLR1, AC087797.5. [file 12870_2015_532_MOESM1_ESM.pdf]

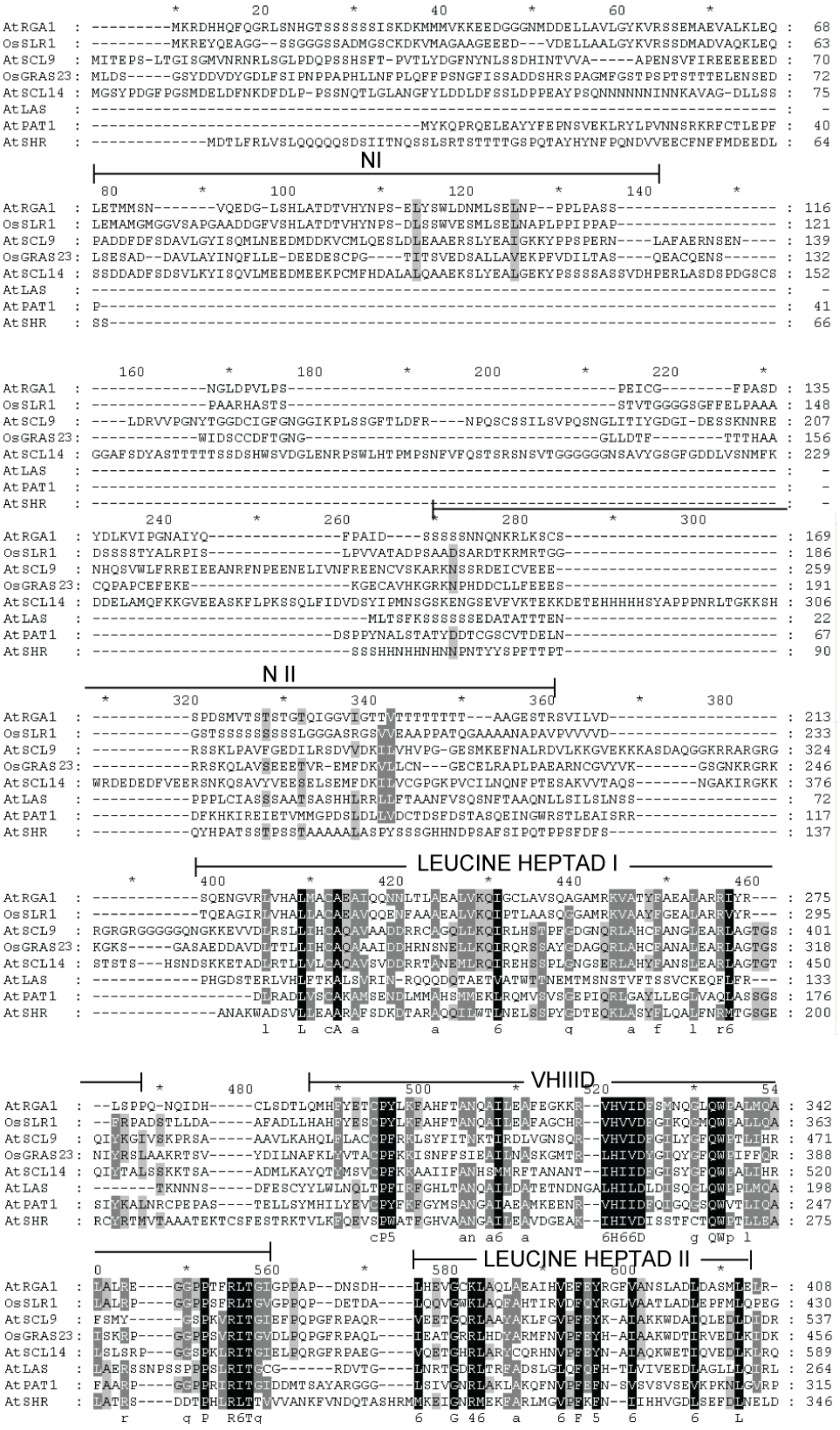

NI
N II
LEUCINE HEPTAD I
VHIID
LEUCINE HEPTAD II
AtRGA1
OsSLR1
AtSCL9
OsGRAS23
AtSCL14
AtLAS
AtPAT1
AtSHR

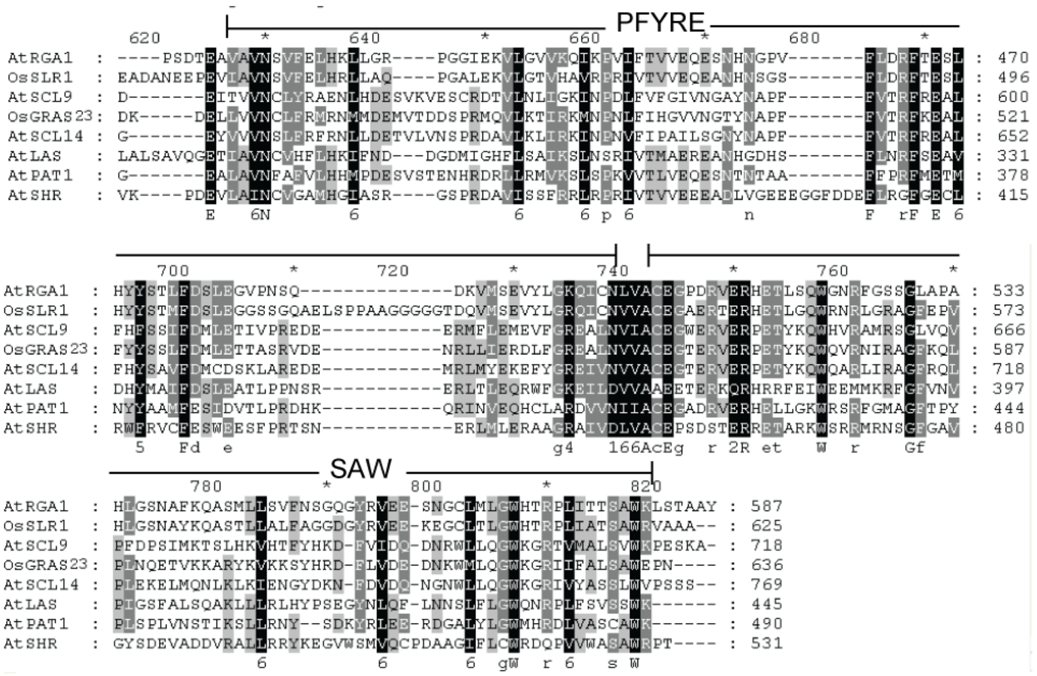

Figure S1. Alignment of deduced amino acids of OsGRAS23 with the well-characterized GRAS family proteins.
