# Supplementary material for: OsGRAS23, a rice GRAS transcription factor gene, is involved in drought stress response through regulating expression of stress-responsive genes
Source: BMC Plant Biol. 2015 Jun 13;15:141. doi: 10.1186/s12870-015-0532-3 (PMC4465154; doi:10.1186/s12870-015-0532-3)
Supplement: Additional file 2: Table S1. — Micro-array analysis of OsGRAS23-overexpressing rice plants. Genes that were up-regulated greater than 2-fold or down-regulated lower than 0.5 in overexpression lines compared with WT are listed. [file 12870_2015_532_MOESM2_ESM.pdf]

Table S1. Micro-array analysis of *OsGRAS23*-overexpressing rice plants. Genes which were up-regulated greater than 2-fold or down-regulated lower than 0.5 in overexpression lines compared with WT were listed.

Probeset ID	Gene Symbol	Fold-Change (OE1-N vs. WT-N)	Fold-Change (OE1-D vs. WT-D)	Fold-Change (WT-D vs. WT-N)	Annotation
Os.8823.1.S1_at	Os03g0115800	59.487	35.164	1.228	Conserved hypothetical protein
Os.10660.1.S1_at	Os07g0162450	30.558	38.046	0.842	Conserved hypothetical protein
Os.10266.1.S1_at	Os03g0629800	24.165	18.122	0.981	Conserved hypothetical protein
Os.46431.1.S1_at	Os01g0537250	10.026	12.857	0.606	Conserved hypothetical protein
Os.2254.1.S1_at	Os07g0129700	7.244	1.703	13.664	OSH15 protein (Homeobox gene)
Os.44418.1.S1_at	Os07g0212400	6.848	3.933	0.915	Conserved hypothetical protein
Os.6863.1.S1_s_at	Os12g0247700	6.126	0.767	8.469	Similar to Jasmonate-induced protein
Os.20501.1.S1_at	Os07g0673900	5.990	3.928	1.349	Hypoxia induced protein conserved region family protein
Os.20614.1.S1_at	Os10g0567900	5.407	5.687	0.573	HAT dimerisation domain containing protein
Os.9273.1.S1_x_at	*Os12g0548650*	4.834	1.314	12.525	Proteinase inhibitor I13 containing protein
Os.54304.1.S1_at	Os04g0173800	4.509	0.944	4.166	Lectin precursor
Os.151.2.S1_at	Os03g0727000	4.508	3.347	1.612	Similar to Homeobox protein OSH1
Os.17112.1.S1_at	Os03g0289800	4.420	1.222	16.545	Similar to Leucoanthocyanidin dioxygenase-like protein
Os.12639.1.A1_at	Os11g0213000	4.415	3.329	1.270	Protein kinase domain containing protein
Os.47730.2.S1_x_at	Os11g0125900	4.143	0.946	2.225	Conserved hypothetical protein
Os.55779.1.S1_at	Os09g0552500	3.894	1.225	2.536	Cupin 1 domain containing protein
Os.4162.1.S1_at	Os05g0129700	3.865	1.766	2.348	KNOX class homeodomain protein

Os.151.1.S1_x_at	Os03g0727000	3.816	1.924	2.523	Similar to Homeobox protein OSH1
Os.55859.1.S1_at	Os04g0308500	3.761	1.246	14.271	unknown
Os.5045.1.S1_at	*Os01g0127600*	3.753	1.149	5.044	Similar to Bowman-Birk type proteinase inhibitor
Os.23219.1.S1_at	Os01g0726700	3.651	2.053	4.360	Conserved hypothetical protein
Os.9836.1.S1_at	Os11g0211800	3.633	1.243	10.935	Conserved hypothetical protein
Os.57548.1.S1_at	Os12g0152000	3.591	1.320	2.646	Similar to Terminal flower 1-like protein
Os.11218.1.S1_at	Os07g0638400	3.560	11.286	1.485	Similar to 1-Cys peroxiredoxin
Os.50413.1.S1_at	Os04g0107600	3.512	1.312	6.472	Arginine decarboxylase
Os.57103.1.S1_at	Os02g0121700	3.401	0.994	8.781	Terpene synthase-like domain containing protein
Os.50352.1.S1_at	Os04g0223500	3.400	1.709	3.274	FAD dependent oxidoreductase family protein
OsAffx.11552.1.S1_at	Os01g0705400	3.321	1.468	0.846	Conserved hypothetical protein
Os.52974.1.S1_at	Os04g0597600	3.288	1.847	3.137	TGF-beta receptor, type I/II extracellular region
Os.49030.1.A1_s_at	Os09g0367700	3.268	2.984	5.550	Similar to GST6 protein
Os.54864.2.S1_at	Os06g0200800	3.218	4.119	1.266	Conserved hypothetical protein
Os.49324.1.S1_at	Os04g0525200	3.214	1.038	2.362	UDP-glucuronosyl/UDP-glucosyltransferase family
OsAffx.26447.3.S1_at	Os04g0516200	3.209	1.630	1.544	Protein of unknown function DUF640 domain
OsAffx.19820.1.S1_at	Os12g0441300	3.188	1.609	1.333	Similar to Flavonoid 4'-O-methyltransferase
Os.6450.1.S1_at	Os07g0190000	3.180	0.763	6.731	Similar to 1-deoxy-D-xylulose 5-phosphate synthase
Os.27292.1.A1_at	Os04g0525100	3.122	1.405	6.583	UDP-glucuronosyl/UDP-glucosyltransferase family protein
Os.4223.1.S1_s_at	Os06g0168700	3.115	1.376	2.447	Similar to Prolin rich protein
Os.9169.1.S1_x_at	Os12g0548700	3.100	1.403	5.050	Transferase family protein
Os.52173.1.S1_at	Os12g0458100	3.055	1.109	2.684	Transferase family protein
Os.12713.1.S2_a_at	Os01g0124000	3.049	1.127	2.910	Similar to Bowman Birk trypsin inhibitor
OsAffx.16673.1.S1_s_at	Os07g0604700	3.045	3.647	1.734	B12D family protein
Os.26820.1.A1_s_at	Os08g0167800	2.985	1.055	1.375	Terpenoid cylases/protein prenyltransferase

Os.15171.1.S1_s_at	Os12g0559200	2.876	0.990	2.933	Lipoxygenase
OsAffx.32261.1.A1_at	Os01g0881700	2.868	2.588	1.256	Similar to Apocytochrome f precursor
OsAffx.30784.1.S1_at	Os11g0117600	2.832	1.639	2.459	Similar to WRKY transcription factor 50
Os.47601.1.A1_at	Os12g0228700	2.811	1.371	3.296	Similar to Jasmonate-induced protein
Os.54358.1.S1_at	Os11g0282700	2.797	1.854	0.977	Homeodomain-like containing protein
Os.53328.1.S1_at	Os09g0543900	2.761	2.066	2.030	Transferase family protein
Os.49726.1.S1_at	Os11g0152500	2.744	1.460	1.506	PEBP family protein
Os.11510.1.S1_s_at	Os09g0491100	2.737	1.951	1.612	Similar to Beta-primeverosidase
Os.264.1.S1_at	Os04g0689000	2.689	1.503	2.723	Similar to Peroxidase
Os.11812.1.S1_at	Os02g0719600	2.684	1.118	6.043	SAM dependent carboxyl methyltransferase family
OsAffx.30784.1.S1_s_at	Os11g0117600	2.660	1.766	1.907	WRKY transcription factor 50
Os.29056.1.S2_at	Os01g0881700	2.628	2.504	1.444	Conserved hypothetical protein
Os.2289.1.S1_at	Os12g0582700	2.622	0.933	5.074	Cytochrome P450 family protein
Os.46471.1.S1_at	Os10g0347000	2.604	1.530	1.234	X8 domain containing protein
OsAffx.2978.2.S1_s_at	Os05g0129700	2.599	1.141	2.098	KNOX class homeodomain protein
Os.405.1.S1_a_at	Os12g0559200	2.589	0.998	2.491	Lipoxygenase
Os.15801.1.S1_x_at	Os11g0126800	2.577	1.989	0.835	Nucleoside phosphatase GDA1/CD39 family protein
Os.12642.1.S1_at	Os08g0540400	2.576	1.723	3.040	Calcium-dependent protein kinase
Os.6645.1.S1_s_at	Os07g0432201	2.572	1.589	1.906	Conserved hypothetical protein
Os.53667.1.S1_at	Os06g0546500	2.561	1.557	1.892	Class III peroxidase GvPx2b
Os.50805.1.S1_at	Os08g0297800	2.555	1.061	2.780	Sulfotransferase family protein
Os.7095.1.S1_at	Os04g0581100	2.550	1.528	23.425	Isopenicillin N synthase family protein
OsAffx.26673.1.S1_at	Os05g0100100	2.542	1.453	1.036	Conserved hypothetical protein
Os.54959.1.S1_at	Os03g0671800	2.540	1.844	1.868	Basic helix-loop-helix dimerisation region bHLH
Os.49663.1.S1_at	Os02g0602000	2.525	1.235	2.371	Remorin, C-terminal region domain

Os.15830.1.S1_at	Os05g0475400	2.510	2.563	1.503	Alanine:glyoxylate aminotransferase-like
Os.55259.1.S1_at	Os04g0395800	2.508	0.760	6.765	ZIM domain containing protein
Os.4618.1.S1_at	Os01g0510200	2.477	2.457	1.254	Conserved hypothetical protein
Os.44546.1.A1_x_at	Os07g0129700	2.445	1.572	4.292	OSH15 protein (Homeobox gene
Os.37729.1.S1_s_at	Os01g0124200	2.440	1.241	4.928	Bowman Birk trypsin inhibitor
Os.54671.1.S1_at	Os08g0277200	2.434	1.278	8.254	Cinnamoyl-CoA reductase
Os.18572.1.S1_at	Os04g0613000	2.428	1.544	0.757	Zinc transporter ZIP1
Os.14882.1.S1_at	Os12g0116600	2.426	1.137	2.362	Transcription factor
Os.4620.1.S1_at	Os06g0318533	2.412	2.629	0.882	Conserved hypothetical protein
OsAffx.14656.1.S1_at	Os05g0214300	2.409	1.011	0.890	MtN3 and saliva related transmembrane protein
Os.53604.1.S1_at	Os03g0738600	2.406	1.337	3.250	Lipoxygenase L-2
Os.50104.1.S1_at	Os06g0604300	2.397	1.066	3.511	Phospholipase D
Os.9421.1.S1_at	Os10g0191300	2.388	1.413	1.431	PR-1a pathogenesis related protein
Os.52178.1.S1_at	Os11g0604400	2.368	1.649	1.052	Conserved hypothetical protein
OsAffx.3569.1.S1_at	Os03g0718800	2.342	1.194	4.310	Physical impedance induced protein
Os.50053.1.A1_at	Os07g0154100	2.305	0.686	21.813	Similar to Viviparous-14
Os.4163.1.S1_at	Os01g0302500	2.300	0.774	3.005	Knotted1-type homeobox protein OSH6
Os.26849.1.S1_at	Os08g0168000	2.269	0.995	1.490	Terpenoid cylases
OsAffx.30149.1.S1_s_at	Os09g0531600	2.269	1.429	1.272	DUF702 family protein
Os.49799.1.S1_at	Os08g0248100	2.260	1.005	3.753	Protein kinase-like domain containing protein
OsAffx.13633.1.S1_at	Os04g0121100	2.255	1.204	1.079	Peptidase S8 and S53, subtilisin, kexin, sedolisin
Os.5318.1.S1_a_at	Os10g0576600	2.241	1.500	2.204	Tetratricopeptide region domain containing protein
OsAffx.17872.1.S1_s_at	Os09g0409100	2.226	1.826	1.246	Conserved hypothetical protein
OsAffx.15538.1.S1_at	Os06g0320200	2.225	0.937	1.254	Glycoside hydrolase
Os.6219.1.S1_at	Os04g0536300	2.224	1.029	1.531	Yabby15 protein
Os.10083.1.S1_at	Os04g0592600	2.222	1.022	2.361	Conserved hypothetical protein

OsAffx.3569.1.S1_x_at	Os03g0718800	2.213	1.153	4.360	Conserved hypothetical protein
Os.51532.1.S1_at	Os12g0154700	2.207	2.271	2.463	Nectarin 1 precursor
Os.11510.1.S1_at	Os09g0491100	2.206	2.168	1.038	Conserved hypothetical protein
Os.52874.1.S1_at	Os08g0112900	2.203	1.085	1.519	Beta-primeverosidase
Os.8673.1.S1_at	Os11g0306400	2.197	1.522	1.585	Herbicide safener binding protein
OsAffx.19456.1.A1_at	Os12g0113600	2.196	3.417	0.977	Conserved hypothetical protein
Os.10435.1.S1_at	Os03g0836800	2.194	1.507	1.704	IAA-amino acid hydrolase 1
Os.1564.1.S1_at	Os01g0327500	2.183	1.001	1.238	Conserved hypothetical protein
OsAffx.14326.1.S1_s_at	Os04g0574200	2.178	1.562	0.933	Beta-Ig-H3/fasciclin domain containing protein
Os.2867.1.A1_at	Os11g0684000	2.165	0.886	6.440	Transcription factor MYB21
Os.55363.1.S1_at	Os07g0592100	2.163	1.040	7.340	Alcohol dehydrogenase-like protein
Os.22935.1.S1_at	Os05g0211100	2.162	1.144	8.215	Cytochrome P450-like protein
Os.21260.1.S1_at	Os03g0184100	2.143	1.186	1.635	Conserved hypothetical protein
Os.50790.1.S1_at	Os03g0733600	2.138	1.342	1.021	SSXT family protein
Os.47815.1.A1_at	Os12g0484900	2.137	1.057	0.764	Growth-regulating factor 7
Os.3697.1.S1_at	Os06g0112100	2.136	1.252	1.959	Conserved hypothetical protein
OsAffx.7246.1.S1_x_at	Os11g0483000	2.132	0.951	3.585	Cytochrome P450 family protein
Os.29290.1.S1_at	Os01g0183300	2.132	1.364	4.429	Endonuclease
Os.23221.1.A1_at	Os12g0289600	2.130	1.956	0.583	Conserved hypothetical protein
OsAffx.6015.1.S1_at	Os08g0434500	2.121	1.871	1.068	Conserved hypothetical protein
Os.5124.1.S1_at	Os05g0573300	2.116	0.988	7.006	CTP synthase 1
Os.48082.1.S1_at	Os09g0417800	2.107	0.923	3.594	WRKY transcription factor 62
Os.54936.1.S1_at	Os03g0790500	2.107	0.827	13.109	Esterase/lipase/thioesterase domain containing
Os.9661.1.S1_at	Os03g0139500	2.105	1.201	1.390	CONSTANS-like protein CO9
OsAffx.23247.1.S1_at	Os01g0248701	2.100	0.787	3.927	Polyprenyl synthetase family protein
Os.467.1.S1_a_at	Os01g0609300	2.084	1.385	5.683	PDR-like ABC transporter
Os.19971.1.S1_at	Os05g0498800	2.081	2.053	0.876	Transposase

OsAffx.5147.1.S1_at	Os06g0700650	2.078	0.970	5.048	ATPase, P-type cation/H+ transporter
Os.53795.1.S1_at	Os03g0187400	2.076	1.064	4.179	Zinc finger, FYVE/PHD-type domain
Os.38638.1.S1_at	Os06g0142200	2.070	1.925	3.896	Early nodulin
Os.32298.1.S1_at	Os03g0187800	2.063	2.086	1.666	DUF250 domain
OsAffx.32315.1.A1_at	Os08g0252200	2.061	1.271	1.653	Photosystem II P680 chlorophyll Aapoprotein (CP-47 protein)
Os.54672.1.S1_at	Os03g0318400	2.059	1.828	3.109	Peptidase A1
Os.51382.1.S1_at	Os01g0954100	2.050	1.307	2.327	Conserved hypothetical protein
OsAffx.14888.1.S1_at	Os05g0368000	2.050	0.659	3.096	Conserved hypothetical protein
Os.11770.1.S1_at	Os07g0677400	2.050	1.525	1.139	Peroxidase
Os.56950.1.S1_at	Os05g0163900	2.047	2.073	6.075	helix-loop-helix dimerisation region bHLH
Os.14532.1.S1_at	Os12g0113600	2.037	2.105	1.574	Conserved hypothetical protein
Os.26927.1.S1_at	Os07g0637000	2.035	1.081	1.697	CBL-interacting serine/threonine-protein (SOS2-like protein kinase PKS13)
Os.20238.1.S1_s_at	Os02g0584200	2.033	1.529	3.274	Conserved hypothetical protein
Os.23207.1.S1_at	Os08g0137800	2.021	1.908	2.719	Conserved hypothetical protein
Os.16282.1.A1_at	Os06g0567200	2.017	1.385	0.504	Cupredoxin domain containing protein
OsAffx.26801.1.S1_x_at	Os05g0186900	2.014	0.847	0.796	Conserved hypothetical protein
Os.36264.1.S1_x_at	Os01g0739700	2.013	1.296	1.509	Glycoside hydrolase
Os.32459.2.S1_x_at	Os01g0123900	2.007	0.848	2.978	Bowman-Birk type proteinase inhibitor
Os.14153.3.S1_x_at	Os05g0111300	2.004	1.362	1.875	B22EL8 protein
Os.54966.1.S1_at	Os06g0691400	2.001	1.152	5.194	IAA-amino acid conjugate hydrolase-like
OsAffx.11145.1.S1_s_at	Os01g0318400	2.001	1.078	0.444	Conserved hypothetical protein
Os.19229.1.S1_a_at	Os03g0639300	0.499	1.105	0.255	Conserved hypothetical protein
Os.51117.1.S1_at	Os08g0359200	0.498	0.913	0.228	Lipid phosphate phosphatase 2
OsAffx.28017.1.S1_at	Os06g0601600	0.498	0.953	0.344	Basic-leucine zipper (bZIP) transcription factor domain containing protein

Os.2019.1.S1_at	Os01g0700100	0.497	1.373	0.540	MtN3 and saliva related transmembrane protein
Os.9900.1.S1_at	Os06g0587401	0.496	0.970	1.248	hypothetical protein
Os.54440.1.S1_at	Os04g0623200	0.496	1.133	0.184	Conserved hypothetical protein
Os.27810.1.S1_at	Os07g0162600	0.495	1.052	0.516	hypothetical protein
Os.14125.1.S1_at	Os09g0522200	0.495	1.277	0.058	DRE-binding protein 1A
Os.10972.1.S1_at	Os01g0878700	0.495	1.136	0.223	Amino acid/polyamine transporter II family protein
Os.15284.1.S1_at	Os07g0680600	0.495	1.022	0.328	Conserved hypothetical protein
OsAffx.31986.1.S1_at	Os12g0510750	0.494	0.738	0.921	Conserved hypothetical protein
Os.11253.1.S1_at	Os01g0850900	0.493	1.111	1.442	SOUL heme-binding protein family protein
Os.49619.1.S1_at	Os03g0315400	0.492	0.951	0.339	Similar to Typical P-type R2R3 Myb protein
Os.33741.1.S1_at	Os07g0224900	0.492	1.178	0.343	WD40-like domain containing protein
Os.27430.1.S1_at	Os08g0183900	0.492	1.067	0.308	NAD-dependent epimerase/dehydratase family protein
OsAffx.15319.1.S1_at	Os06g0187500	0.492	0.845	0.363	UDP-glucuronosyl/UDP-glucosyltransferase family
Os.26957.1.A1_a_at	Os08g0200600	0.491	0.755	0.714	Similar to NAC-domain containing protein 21/22
Os.40021.1.S1_a_at	Os03g0161900	0.491	0.649	10.933	Similar to Heat shock factor 1
Os.12206.1.S1_at	Os07g0124100	0.489	0.950	0.726	Phytosulfokines 4 precursor
Os.48724.1.S1_at	Os08g0401500	0.488	1.050	0.337	Similar to HOTHEAD protein precursor
Os.32374.2.S1_at	Os01g0719250	0.488	1.215	0.236	Conserved hypothetical protein
Os.51273.1.S1_at	Os02g0609000	0.488	0.840	0.451	Conserved hypothetical protein
Os.1411.1.S1_at	Os01g0206700	0.486	1.241	0.210	Similar to Serine/threonine protein kinase
Os.14885.1.S1_at	Os05g0510100	0.485	1.022	1.760	unknown function DUF567 family protein
Os.53428.1.S1_at	Os09g0433800	0.484	1.082	0.139	Senescence-associated protein SAG102
Os.4775.1.S1_at	Os01g0136200	0.484	0.843	0.850	16.9 kDa class I heat shock protein
Os.25639.1.S1_at	Os04g0352400	0.482	0.714	11.040	Peptidylprolyl isomerase
Os.32596.3.S1_x_at	Os01g0944100	0.482	1.147	2.564	Conserved hypothetical protein

Os.7665.1.S1_at	Os04g0657500	0.481	1.182	0.128	Lipase, class 3 family protein
Os.4679.1.S1_at	Os06g0133200	0.481	1.194	0.251	Conserved hypothetical protein
Os.12110.1.S1_at	Os11g0118300	0.480	1.812	0.748	NPH3 domain containing protein
OsAffx.4280.2.A1_at	Os05g0181700	0.480	1.141	0.379	Conserved hypothetical protein
Os.7879.2.S1_at	Os04g0659100	0.480	0.973	0.525	Glutamine synthetase shoot isozyme
Os.15671.1.A2_at	Os11g0644800	0.479	1.416	0.210	Aminotransferase
OsAffx.4677.1.S1_s_at	Os05g0582000	0.478	0.961	0.351	Conserved hypothetical protein
Os.49287.1.S1_x_at	Os04g0542000	0.478	1.326	0.215	HAT dimerisation domain containing protein
Os.34087.2.A1_at	Os01g0650100	0.476	1.389	0.206	unknown
Os.32177.1.S1_at	Os05g0586600	0.475	1.053	1.612	Plastid sigma factor SIG5
Os.26838.1.S1_at	Os04g0592500	0.475	1.081	0.362	Phosphoenolpyruvate carboxykinase (ATP) family protein
Os.8468.1.S1_at	Os12g0438400	0.475	1.511	0.157	Hypothetical protein
Os.27755.1.S1_at	Os07g0539900	0.475	1.180	0.511	Beta-1,3-glucanase-like protein
Os.26811.1.A1_at	Os06g0599200	0.474	0.807	0.707	Cytochrome P450 family protein
Os.8044.1.S1_at	Os02g0206100	0.473	1.293	0.253	UDP-glucuronosyl/UDP-glucosyltransferase family protein
Os.1977.1.S1_a_at	Os01g0114300	0.473	1.085	0.373	Protein kinase-like domain containing protein
Os.15641.1.S1_at	Os01g0965900	0.468	1.166	0.545	Conserved hypothetical protein
Os.49564.1.S1_at	Os04g0412100	0.468	0.979	0.483	Conserved hypothetical protein
Os.27659.1.S1_x_at	Os01g0179800	0.467	0.974	0.156	TMS membrane protein
Os.9276.1.S1_s_at	Os07g0175300	0.467	1.295	0.204	Conserved hypothetical protein
Os.37051.1.S1_at	Os06g0591400	0.465	1.195	0.265	Conserved hypothetical protein
Os.9138.1.S1_at	Os09g0535500	0.465	0.970	0.295	RING-H2 finger protein ATL1R
Os.10556.1.S1_at	Os02g0258800	0.464	0.930	0.200	Conserved hypothetical protein
Os.7507.1.S1_at	Os10g0118200	0.463	1.228	0.248	O-methyltransferase ZRP4
Os.57354.1.S1_at	Os04g0660000	0.460	1.133	0.333	Conserved hypothetical protein

Os.11150.1.S1_at	Os03g0439700	0.459	1.043	0.323	unknown function DUF1230 family protein
Os.11046.1.S1_at	Os06g0592500	0.458	0.660	27.302	Similar to Ethylene-responsive transcriptional coactivator
Os.37837.1.A1_at	Os01g0904200	0.458	1.493	0.202	protein kinase-like domain containing protein
Os.7007.1.S1_at	Os02g0213100	0.457	0.844	0.551	Conserved hypothetical protein
Os.26552.1.S1_at	Os10g0539700	0.456	1.541	0.267	Conserved hypothetical protein
Os.49287.1.S1_at	Os04g0542000	0.456	1.633	0.186	HAT dimerisation domain containing protein
Os.56880.1.S1_at	Os01g0542700	0.455	1.477	0.579	Eukaryotic transcription factor
Os.56275.1.S1_x_at	Os10g0560400	0.454	1.560	0.512	CONSTANS-like protein CO9
Os.55886.1.S1_at	Os01g0765600	0.453	0.950	0.228	EF-Hand type domain containing protein
Os.25171.1.A1_at	Os11g0695850	0.452	0.965	0.277	EGF-like, type 3 domain containing protein
OsAffx.23090.2.S1_x_at	Os01g0117500	0.451	1.003	0.451	LRK14
Os.18378.1.S1_s_at	Os10g0335000	0.451	0.846	0.562	Conserved hypothetical protein
Os.35559.1.S1_at	Os05g0495700	0.446	0.892	0.377	Glycerol-3-phosphate dehydrogenase-like protein
Os.10583.1.S1_s_at	Os06g0141200	0.446	0.784	0.297	RNA-binding protein EWS
Os.11945.1.S1_at	Os08g0386200	0.445	1.025	0.512	WRKY transcription factor 69
OsAffx.5232.1.S1_s_at	Os07g0158400	0.444	1.249	0.302	GCK domain containing protein
Os.55270.1.S1_s_at	Os04g0634800	0.443	1.022	0.486	Conserved hypothetical protein
Os.13999.1.S1_at	Os02g0178100	0.443	1.517	0.394	CCT domain containing protein
Os.12082.1.S1_at	Os04g0469000	0.440	1.308	0.777	Heavy metal transport/detoxification protein domain containing protein
Os.17762.1.S1_at	Os06g0494400	0.438	1.122	0.361	Multi antimicrobial extrusion protein MatE family protein
Os.56184.1.S1_at	Os04g0480200	0.438	1.358	0.424	Fibronectin
OsAffx.14410.1.S1_s_at	Os04g0635000	0.437	0.837	0.364	Conserved hypothetical protein
Os.7017.1.S1_at	Os01g0364800	0.433	0.944	0.487	EGF-like calcium-binding domain containing protein

Os.6976.2.A1_at	Os09g0304800	0.430	0.829	0.336	Conserved hypothetical protein
Os.6651.1.S1_at	Os07g0630400	0.429	1.112	0.439	Ribonuclease T2 family protein
Os.12007.1.S1_at	Os03g0364400	0.428	1.351	0.225	Phytosulfokine receptor-like protein
Os.51583.1.S1_at	Os12g0420400	0.425	1.220	0.303	Photosystem I reaction center subunit XI
Os.28964.1.S1_at	Os01g0613800	0.424	1.142	0.770	Peptidase C1A, papain family protein
Os.5816.1.S1_at	Os09g0522000	0.421	1.066	0.045	Similar to CBF-like protein
Os.4921.1.S1_at	Os12g0222300	0.421	0.656	0.401	Hypothetical protein
Os.51022.1.A1_at	Os09g0471400	0.421	1.038	0.275	Protein kinase-like domain containing protein
Os.11602.1.S1_at	Os09g0553900	0.420	1.446	0.112	Conserved hypothetical protein
Os.53728.1.S1_at	Os09g0482640	0.420	0.744	0.353	EGF-like calcium-binding domain containing protein
Os.50969.1.S1_at	Os03g0664400	0.419	1.169	0.304	Expressed protein
Os.14101.3.S1_at	Os12g0137100	0.419	1.097	0.224	Annexin, type VII family protein
Os.11244.3.S1_x_at	Os06g0133000	0.419	1.290	0.332	Granule-bound starch synthase I
Os.33350.1.S1_x_at	Os07g0407700	0.416	1.114	0.250	Hypothetical protein
Os.53177.1.S1_at	Os06g0206900	0.415	0.942	1.045	Conserved hypothetical protein
Os.18814.1.S1_at	Os11g0597700	0.408	1.558	0.259	Conserved hypothetical protein
Os.28124.1.S1_at	Os10g0416500	0.406	1.163	0.555	Similar to Chitinase 1 precursor
OsAffx.25073.1.S1_x_at	Os03g0228200	0.404	1.114	0.061	Conserved hypothetical protein
Os.26900.1.A1_at	Os09g0339000	0.403	0.828	0.494	Protein kinase-like domain containing protein
Os.4863.1.S1_at	Os01g0631200	0.402	1.575	0.126	Similar to Uroporphyrinogen III methyltransferase
Os.49503.1.S1_at	Os03g0388500	0.402	1.023	0.579	Similar to Anther ethylene-upregulated protein ER1
Os.52820.1.S1_at	Os11g0416900	0.400	0.669	0.458	ABC transporter related domain containing protein
Os.51145.1.S1_at	Os11g0566800	0.396	0.628	0.292	Similar to Bibenzyl synthase
Os.1478.1.S1_at	Os03g0307300	0.396	1.374	0.306	Nicotianamine synthase 1
Os.16245.1.S1_at	Os03g0293000	0.394	0.737	9.031	Thioredoxin fold domain containing protein

Os.18955.1.S1_at	Os06g0728700	0.393	0.954	2.497	Homeodomain-like containing protein
OsAffx.24280.2.S1_at	Os07g0178700	0.385	0.950	0.474	Similar to Low molecular mass early light-inducible protein HV90
Os.23635.1.S1_at	Os01g0847100	0.384	1.363	0.382	Conserved hypothetical protein
OsAffx.17798.1.S1_at	Os09g0369400	0.382	0.890	0.082	Similar to Trehalose-6-phosphate phosphatase
Os.27036.1.S1_at	Os04g0307500	0.382	0.887	0.281	EGF-like calcium-binding domain containing protein
Os.20531.1.S1_at	Os04g0632600	0.381	0.619	0.288	Similar to Receptor-like protein kinase 5
Os.15854.1.S1_at	Os07g0543300	0.381	1.574	0.234	Conserved hypothetical protein
Os.10751.1.S1_at	Os10g0560400	0.379	1.541	0.337	CONSTANS-like protein CO9
Os.4717.1.S1_at	Os08g0468100	0.379	1.314	0.025	Nitrate reductase [NADH] 1
OsAffx.13282.1.S1_s_at	Os03g0609500	0.375	0.976	0.224	LOB domain protein 39
Os.47474.1.S1_at	Os06g0650900	0.372	0.664	1.562	Heat shock protein DnaJ family protein
Os.11039.1.S1_s_at	Os04g0107900	0.372	0.374	23.896	Similar to Heat shock protein 80
Os.12112.1.S1_at	Os01g0823600	0.368	1.394	0.577	Conserved hypothetical protein
Os.52451.1.A1_at	Os09g0457900	0.366	0.663	0.129	Similar to AP2 domain containing protein RAP2.6
Os.33350.1.S1_at	Os07g0407700	0.364	1.206	0.237	Hypothetical protein
Os.50830.1.S1_at	Os09g0414500	0.359	0.933	0.115	Similar to ZF-HD homeobox protein
Os.21524.1.S1_at	Os10g0149000	0.357	0.305	0.870	unknown function DUF1210 family protein
Os.28427.1.S2_a_at	Os05g0217800	0.355	1.080	0.364	BURP domain containing protein
Os.54146.1.S1_at	Os02g0770800	0.354	1.517	0.090	Similar to Nitrate reductase
OsAffx.11989.2.S1_s_at	Os02g0206700	0.350	1.337	0.294	UDP-glucuronosyl/UDP-glucosyltransferase family
Os.9445.1.S1_at	Os04g0635100	0.350	1.050	0.169	Conserved hypothetical protein
Os.17916.1.S1_at	Os05g0217700	0.349	1.186	0.051	Conserved hypothetical protein
Os.30376.1.S1_at	Os01g0111700	0.349	1.667	0.660	Conserved hypothetical protein
Os.46574.1.S1_at	Os10g0570200	0.346	0.850	0.774	Similar to RIR1b protein precursor
Os.6976.1.S1_s_at	Os03g0126000	0.344	0.325	0.976	Similar to Phosphorybosyl anthranilate

					transferase 1
Os.55740.1.S1_at	Os06g0671300	0.340	0.491	0.613	Cytochrome P450 family protein
Os.28011.1.S1_at	Os06g0493100	0.338	0.578	0.658	Conserved hypothetical protein
Os.10280.1.A1_at	Os02g0137700	0.333	1.420	0.806	Conserved hypothetical protein
Os.35583.1.S1_at	Os01g0681800	0.322	1.136	0.387	Conserved hypothetical protein
Os.45516.1.S1_at	Os01g0117200	0.320	1.079	0.274	Similar to ARK protein
Os.23327.2.S1_a_at	Os10g0180800	0.307	0.533	0.156	EGF domain containing protein
Os.52627.1.S1_at	Os08g0351300	0.289	0.776	0.316	Hypothetical protein
OsAffx.15178.1.S1_s_at	Os05g0579600	0.279	1.283	0.091	Homeodomain-like containing protein
Os.6764.1.S1_at	Os04g0635600	0.272	0.818	0.085	Conserved hypothetical protein
Os.171.1.S1_at	Os01g0660200	0.250	1.183	0.679	Acidic class III chitinase OsChib3a precursor
Os.15516.1.S1_at	Os04g0370900	0.245	0.671	0.503	Conserved hypothetical protein
Os.26732.1.S1_at	Os01g0175700	0.245	0.934	0.261	UDP-glucuronosyl/UDP-glucosyltransferase family protein
Os.57519.1.S1_x_at	Os01g0246400	0.244	1.000	1.145	hypothetical protein
Os.15285.1.S1_a_at	Os01g0369000	0.238	0.238	0.856	Similar to Cullin-1
Os.35456.1.S1_at	Os06g0582600	0.238	0.672	0.324	Similar to Cysteine proteinase
Os.9191.1.S1_s_at	Os01g0246400	0.231	1.110	1.059	Similar to Low molecular mass early light-inducible protein HV90
Os.6867.1.S1_x_at	Os12g0628600	0.231	0.687	0.790	Similar to Thaumatin-like pathogenesis-related
Os.12430.1.S1_at	Os07g0142100	0.219	3.981	0.404	Conserved hypothetical protein
Os.39294.1.S1_at	Os09g0471500	0.219	0.853	0.191	Protein kinase-like domain containing protein
Os.57142.1.S1_at	Os06g0671600	0.217	0.311	0.601	Conserved hypothetical protein
OsAffx.30449.1.S1_at	Os10g0335000	0.190	0.807	0.227	Conserved hypothetical protein
Os.9762.2.S1_at	Os08g0355600	0.142	0.700	0.224	unknown
Os.53763.1.A1_at	Os06g0581500	0.076	1.170	0.050	Protein kinase-like domain containing protein
