# Supplementary material for: OsGRAS23, a rice GRAS transcription factor gene, is involved in drought stress response through regulating expression of stress-responsive genes
Source: BMC Plant Biol. 2015 Jun 13;15:141. doi: 10.1186/s12870-015-0532-3 (PMC4465154; doi:10.1186/s12870-015-0532-3)
Supplement: Additional file 3: Table S2. — Primers used in this research. [file 12870_2015_532_MOESM3_ESM.pdf]

Table S2. Primers used in this research

Primer name	Primer sequence（5'-3'）	Description
**Transgenic plant**		
GRAS-XbaI	GCTCTAGAATGTTGGATTCTGGTTCTTATG	overexresssion vector
GRAS-BstEII	GGTCACCCTAGTTTGGTTCCCATGC	
GRAS1PGf1	CCCAAGCTTCGCATGAACGATGAATAAC	promoter-*GUS* vector
GRAS1PGr1	CGGGATCCGACTCTTGAATCTTCGAAC	
GRASGf	GCTCTAGAATGTTGGATTCTGGTTCTTATGATG	GFP vector
GRASGr	CGGGATCCGTTTGGTTCCCATGCTGAAAGAGC	
**Transactivation activity**		
attB GRASf	AAAAAGCAGGCTCGATGTTGGATTCTGGT	full length
attB GRASr	AGAAAGCTGGGTCTAGTTTGGTTCCCAT	
attB GRASQC1r	AGAAAGCTGGGTCTATAGGGTAGTGAGATCAAC	N1-264
attB GRASQN1f	AAAAAGCAGGCTCGCTCATTCATTGTGCC	C265-636
attB GRASN2r	AGAAAGCTGGGTCTAGAACGTATCAAGCAATCC	N1-149
attB GRASN3f	AAAAAGCAGGCTCGACAACCACACATGCC	N150-264
**Realtime PCR**		
ActF	CTTCCTCATGCCATCCTGC	*Actin*
ActR	GCAAGCTTCTCCTTGATGTCC	
GRASF	GCCCGTATGTTCAATGTTCCA	*OsGRAS23*
GRASR	GTCATCTGTCACCATTTCGTCC	
62450 F	GTGATACCTACACCAAAATCTACGG	*Os07g0162450*
62450 R	GCTTGACAGACTGGAAGGCTACT	
29800 F	CAGGGTCTATCATTCGGGTTG	*Os03g0629800*
29800 R	GCTTGACAGACTGGAAGGCT	
37250F	CCAAAGATGGTGGCGAA	*Os01g0537250*
37250R	GCAGTGCTGGCGTGGAT	
67700 F	TGGTGCTCCTGGACTTGTG	*Os09g0367700*
67700 R	CGGCGACTCCTTCTTCTTC	
38400 F	GGTGGTGCTCCTGGACTTG	*Os07g0638400*
38400 R	CGGCGACTCCTTCTTCTTCT	
89000 F	CTTCGACAACGCCTACTACATC	*Os04g0689000*
89000R	GCACATTGCTTAGTTTGACCATG	
73900F	TCACAGCAGGCACAAACA	*Os07g0673900*
73900R	AGTCAGCCACAGCGTCCC	
47700F	TGTTCAGGGCAACCAGAT	*Os12g0247700*
47700R	ACCTGAAGCGTGGACCC	
48650F	GAGGAGGAGCGGAAGACG	*Os12g0548650*
48650R	CGACGAAGATGCGGACG	
89800F	TGTGCAGGAGGGTGATGAG	*Os03g0289800*
89800R	CCGATGTTGACGATGAAGG	
24000F	GCTCTTCCTCCTCGTCCT	*Os01g0124000*
24000R	CCACGGTCTGGATTTCG	
**Yeast one hybrid**		

GRAS Y1F	TCCCCCGGGTATGTTGGATTCTGGTTCTTATG	*OsGRAS23*
GRAS Y1R	CGGGATCCCCTAGTTTGGTTCCCATGCTG	
P2450F	GGAATTCTGAGACGGAGGGAGTAGCTGAGCATGG	
P2450R	TCCCCCGGGGACATGAGAAATCAGATGGTTGCG	
P9800F	GGAATTCAGAGCAAGGAGGTGGCCGACAAGG	
P9800R	TCCCCCGGGAACACCAACTAAGGACAAACC	
P7250F	GGGAATTCTGGAGTGAATCCAGTCGGTTCCAATGG	
P7250R	TCCCCCGGGGTCTCCAGCTTCCATACCGCACCAAC	
P8400F	GGAATTCCCAATGTAACCAAAGAATGAGAAC	
P8400R	TCCCCCGGGATTGCAATTCGCAAATGTTCTGAC	
P9390F	CCGGAATTCAAGCCCCGCATACTCTACA	
P9390R	TCCCCCGGGCGTTCTTGGTACTGGATAGTTAG	
P8030F	CCGGAATTCATTATTCGCAGGCGTTCTTGG	
P8030R	TCCCCCGGGCTAGCACGTCATGCACTCG	
P1890F	CCGGAATTCGAGAAGAGGTGTAGCGGTGGT	
P1890R	TCCCCCGGGCTAAAGCAAGAGGGCTGAAA	
P7670F	CCGGAATTCCCCAGCCGCAGTTTTGA CC	
P7670R	TCCCCCGGGTCTGTTTGTGCCTGCTGTG	
